# Bioactive Phytochemicals from Mulberry: Potential Anti-Inflammatory Effects in Lipopolysaccharide-Stimulated RAW 264.7 Macrophages

**DOI:** 10.3390/ijms22158120

**Published:** 2021-07-29

**Authors:** Dahae Lee, Seoung Rak Lee, Ki Sung Kang, Ki Hyun Kim

**Affiliations:** 1 College of Korean Medicine, Gachon University, Seongnam 13120, Korea; pjsldh@gachon.ac.kr; 2 School of Pharmacy, Sungkyunkwan University, Suwon 16419, Korea; davidseoungrak@gmail.com

**Keywords:** *Morus alba*, inflammation, nitric oxide, nuclear factor kappa B, inducible nitric oxide synthase, cyclooxygenase-2

## Abstract

The fruits of the mulberry tree (*Morus alba* L.), known as white mulberry, have been consumed in various forms, including tea, beverages, and desserts, worldwide. As part of an ongoing study to discover bioactive compounds from *M. alba* fruits, the anti-inflammatory effect of compounds from *M. alba* were evaluated in lipopolysaccharide (LPS)-stimulated mouse RAW 264.7 macrophages. Phytochemical analysis of the ethanol extract of the *M. alba* fruits led to the isolation of 22 compounds. Among the isolated compounds, to the best of our knowledge, compounds **1**, **3**, **5**, **7**, **11**, **12**, and **14–22** were identified from *M. alba* fruits for the first time in this study. Inhibitory effects of 22 compounds on the production of the nitric oxide (NO) known as a proinflammatory mediator in LPS-stimulated RAW 264.7 macrophages were evaluated using NO assays. Western blot analysis was performed to evaluate the anti-inflammatory effects of cyclo(L-Pro-L-Val) (**5**). We evaluated whether the anti-inflammatory effects of cyclo(L-Pro-L-Val) (**5**) following LPS stimulation in RAW 264.7 macrophages occurred because of phosphorylation of IκB kinase alpha (IKKα), IκB kinase beta (IKKβ), inhibitor of kappa B alpha (IκBα), nuclear factor kappa B (NF-κB) and activations of inducible nitric oxide synthase (iNOS), and cyclooxygenase-2 (COX-2). Cyclo(L-Pro-L-Val) (**5**) significantly suppressed phosphorylations of IKKα, IKKβ, IκBα, and NF-κB and activations of iNOS and COX-2 in a concentration-dependent manner. Taken together, these results indicate that cyclo(L-Pro-L-Val) (**5**) can be considered a potential therapeutic agent for the treatment of inflammation-associated disorders.

## 1. Introduction

Inflammation is a complex physiological response to foreign harmful stimuli including particles, toxic compounds, bacteria, and viruses. In the host defense system of macrophages, the regulation of inflammation is involved in immune function [1]. In response to lipopolysaccharide (LPS), macrophages overproduce proinflammatory mediator nitric oxide (NO) and cyclooxygenase (COX-2) to upregulate inflammatory states. NO is synthesized by inducible nitric oxide synthase (iNOS) via the NF-kB signaling pathway. Therefore, inhibition of LPS production and related mechanisms are considered useful targets in research to discover potential anti-inflammatory compounds [2].

The fruits of mulberry tree (*Morus alba* L.) belonging to the family of Moraceae are known as white mulberry, which is popularly consumed in various forms, including tea, beverages, and desserts, worldwide [3]. In Korea and China, where this plant is harvested, the leaves of *M. alba* have been traditionally used as a fodder for silkworms, as well as a medicinal herb to treat diabetes and improve eyesight [4]. The pharmacological activities of *M. alba* have also been actively studied, with reports that the white mulberry has a number of beneficial effects such as antioxidant [5,6,7], anti-microbial [8], anti-obesity [9,10], and anti-inflammatory activities [7].

As part of a continuing research project to discover bioactive natural products, our group has investigated biologically active and structurally interesting compounds from the ethanol extract of fruits of *M. alba* [7,11,12,13,14]. In our previous study, we observed that butyl pyroglutamate identified from the fruits of *M. alba* exhibits protective effects against apoptosis, characteristic of cisplatin-induced kidney cell damage [11]. Our previous research has also shown that indole acetic acid derivative in *M. alba* induces apoptosis via both death receptor-mediated extrinsic pathway and mitochondria-mediated intrinsic pathway [12]. Odisolane, a new oxolane derivative identified from *M. alba*, was found to inhibit the tube formation of human umbilical vein vascular endothelial cells via vascular endothelial growth factor-mediated mechanistic pathway [13]. In addition, loliolide in *M. alba* was demonstrated by our group to protect INS-1 human pancreatic β-cells against apoptosis triggered by streptozotocin [14]. In particular, our recent research on ethanol extract from mulberry fruits reported its inhibitory effect on LPS-stimulated NO production and iNOS expression in a mouse macrophage cell line (RAW 264.7), suggesting its potential for anti-inflammatory effect [7]. In the present study, the ethanol extract of *M. alba* fruits was further investigated to identify potential anti-inflammatory compounds. Phytochemical analysis of the extract of *M. alba* fruits led to the identification of 22 compounds (**1**–**22**). Their structures were determined by detailed analysis of their nuclear magnetic resonance (NMR) spectroscopic and physical data as well as mass spectrometry (MS) data from liquid chromatography–mass spectrometry (LC-MS) analysis. Herein, we report the isolation and structural identification of the compounds (**1**–**22**) and their anti-inflammatory potential in RAW 264.7 cells and basic underlying mechanism of action.

## 2. Results

### 2.1. Isolation and Identification of the Compounds

**Figure 1:**
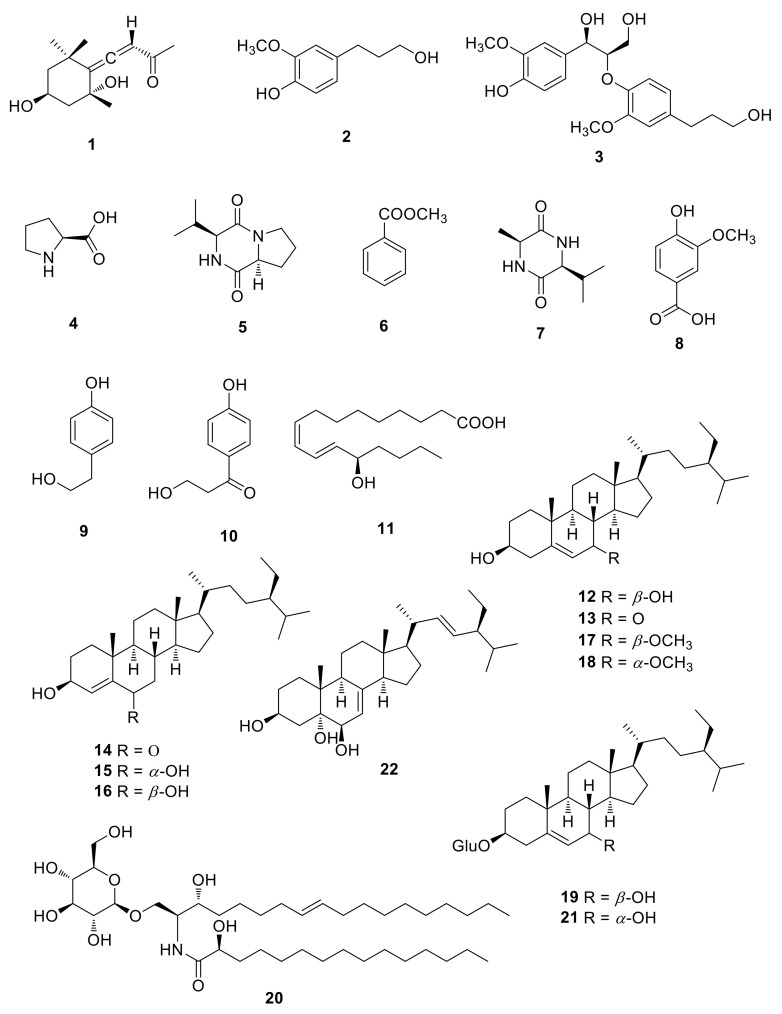
Chemical structures of compounds **1**–**22** isolated from *M. alba* fruits.

Dried and smashed *M. alba* fruits were extracted using 70% aqueous ethanol three times and filtered at room temperature. The filtrate was evaporated in vacuo to obtain a crude ethanol extract. The ethanol extract was solvent-partitioned with hexane, CH_2_Cl_2_, EtOAc, and *n*-BuOH to obtain four fractions. TLC and LC/MS analysis of solvent fractions determined that the CH_2_Cl_2_-soluble fraction could be the main fraction showing a chemical profile with plentiful structural diversity. Phytochemical analysis of the CH_2_Cl_2_ fraction was carried out using repeated column chromatography and preparative/semi-preparative HPLC, which afforded 22 compounds (**1**–**22**) (Figure 1). The structures of the isolated compounds including their absolute configurations were determined to be grasshopper ketone (**1**) [15], dihydroconiferyl alcohol (**2**) [16], *threo*-guaiacylglycerol-*β*-O-4′-dihydroconiferyl alcohol (**3**) [17], L-proline (**4**) [18], cyclo(L-Pro-L-Val) (**5**) [19], methyl benzoate (**6**) [20], cyclo(L-Ala-L-Val) (**7**) [21], vanillic acid (**8**) [21], tyrosol (**9**) [22], 3-hydroxy-1-(4-hydroxyphenyl)-1-propanone (**10**) [23], (*E*,*Z*)-13-hydroxy-9,11-heptadecadienoic acid (**11**) [24], 7*β*-hydroxysitosterol (**12**) [25], 7-ketositosterol (**13**) [26], stigmast-4-en-3*β*-ol-6-one (**14**) [27], (3*β*,6*α*)-stigmast-4-ene-3,6-diol (**15**) [28], stigmast-4-ene-3*β*,6*β*-diol (**16**) [29], (3*β*,7*β*)-7-methoxystigmast-5-en-3-ol (**17**) [30], schleicheol 2 (**18**) [30], 7*β*-hydroxysitosterol 3-O-*β*-D-glucoside (**19**) [25], portulacerebroside B (**20**) [31], 7α-hydroxysitosterol 3-O-*β*-D-glucoside (**21**) [25], and (24*S*)-24-Ethylcholesta-7,22-diene-3*β*,5*α*,6*β*-triol (**22**) [32] by comparing their NMR and physical data such as optical rotation value with those reported in the literature. In particular, the absolute configurations of the amino acid units in **5** were confirmed by the Marfey’s method [33]. The hydrolysis products of **5** were subjected to L-FDAA derivatization and analyzed by electrospray ionization (ESI)-quadrupole-time of flight (Q-TOF)-liquid chromatography (LC)/mass spectrometry (MS) (see Supplementary Materials), which showed that all amino acid residues were L-configurations in **5** (Figures S25–S31). Among the isolated compounds, to the best our knowledge, compounds **1**, **3**, **5**, **7**, **11**, **12**, and **14**–**22** were identified from *M. alba* fruits for the first time in this study.

### 2.2. Effects of Compounds ***1–22*** on Nitric Oxide (NO) Production

**Figure 2:**
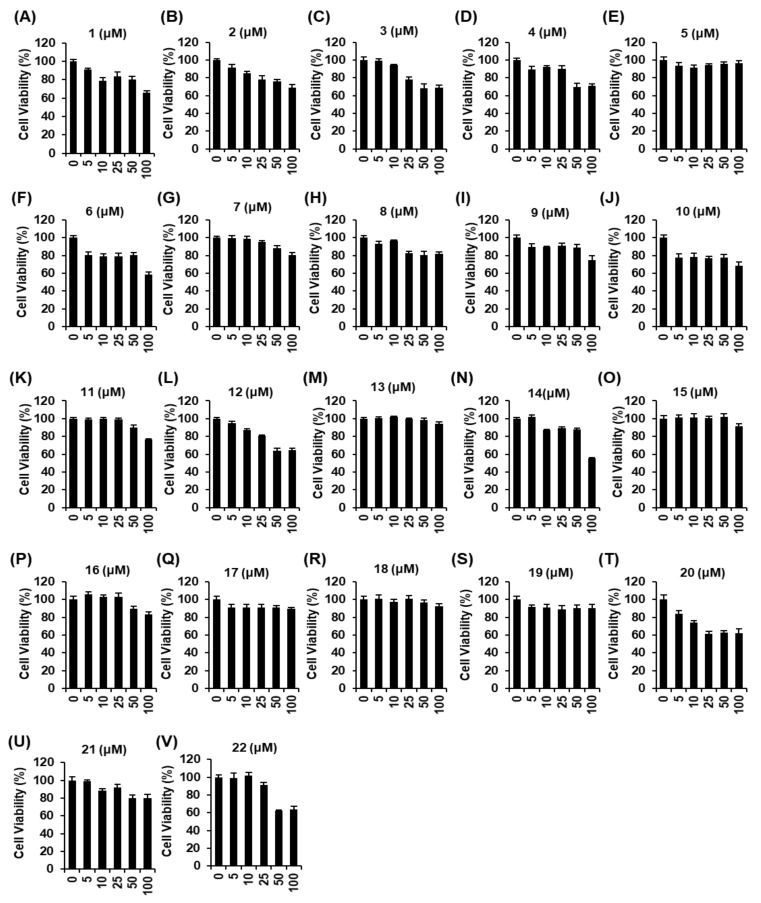
Effects of compounds **1–22** isolated from *M. alba* fruits on the viability of RAW 264.7 mouse macrophages. Effect of (**A**–**V**) compounds **1**–**22**, compared with the control (0 µM), on the viability of RAW 264.7 mouse macrophages for 24 h by MTT assay.

**Figure 3:**
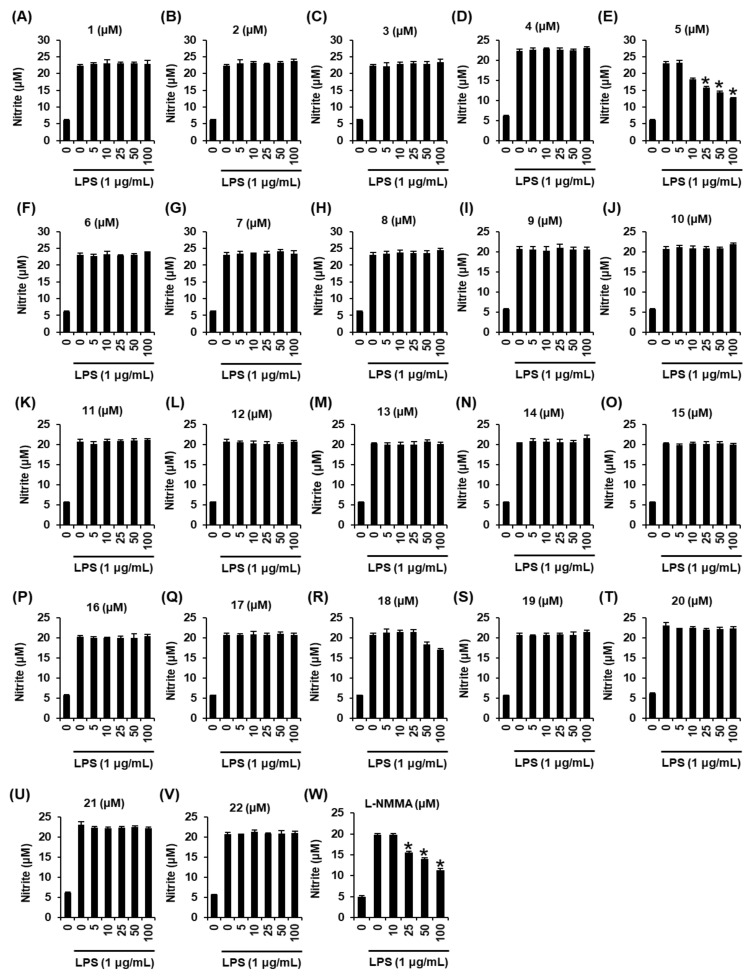
Effects of compounds **1**–**22** isolated from *M. alba* fruits and *N*^G^-methyl-L-arginine acetate salt (L-NMMA) on the nitric oxide (NO) production in RAW 264.7 mouse macrophages treated with lipopolysaccharide (LPS). (**A**–**W**) The effects of compounds **1**–**22** and the nitric oxide (NO) synthase inhibitor L-NMMA in RAW 264.7 mouse macrophages treated with LPS were investigated (mean ± SD, * *p* < 0.05 compared to group treated with 1 µg/mL LPS alone).

The non-toxic dose of compounds **1–22** was determined using a cell viability assay on RAW 264.7 macrophages. Some compounds excluding **5**, **13**, **15**, **16**, **17**, **18**, and **19** were cytotoxic, as cell viability decreased to below 80% (Figure 2). The inhibitory effects of compounds **1–22** on NO production in LPS-activated RAW 264.7 macrophages were investigated. Among these compounds, only compound **5** attenuated nitrite concentration in LPS-activated RAW 264.7 cells. As shown in Figure 3E, compared with the LPS-only group (23.20 ± 0.58 µM), coincubation with compound **5** (25 µM, 50 µM, and 100 µM) and LPS resulted in a 15.81 ± 0.43 µM, 14.31 ± 0.48 µM, and 12.56 ± 0.27 µM, respectively, lower nitrite concentration. As shown in Figure 3W, compared with the LPS-only group (19.75 ± 0.34 µM), coincubation with *N*^G^-methyl-L-arginine acetate salt (L-NMMA) (25 µM, 50 µM, and 100 µM) and LPS resulted in a 15.58 ± 0.27 µM, 13.99 ± 0.29 µM, and 11.33 ± 0.39 µM, respectively, lower nitrite concentration. These results suggest that compound **5** might have potential anti-inflammatory activity that was comparable to L-NMMA in LPS-activated RAW 264.7 cells. In addition, considering the result of cell viability after treatment with compound **5**, its inhibitory effect on nitrite concentration in LPS-activated RAW 264.7 cells was deemed not to be attributable to its cytotoxicity.

### 2.3. Effects of Compound ***5*** on the LPS-Induced Expression of IKKα/β, I-κBα, and NF-κB in RAW 264.7 Mouse Macrophages

**Figure 4:**
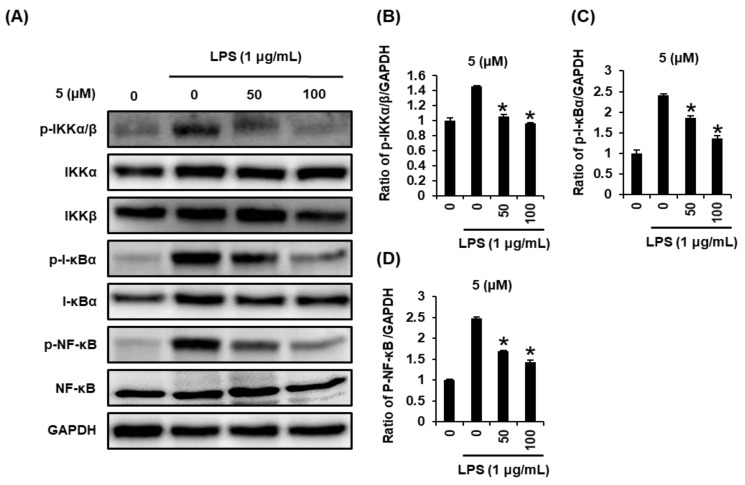
Effects of compound **5** on the expression of IκB kinase alpha and beta (IKKα/β), inhibitor of kappa B alpha (I-κBα), and nuclear factor kappa B (NF-κB) in RAW 264.7 mouse macrophages treated with lipopolysaccharide (LPS). (**A**) Representative Western blots showing protein expressions of IKKα/β, phospho-IKKα/β (p-IKKα/β), I-κBα, phospho-IKKα/β (p-I-κBα), NF-κB, and glyceraldehyde-3-phosphate dehydrogenase (GAPDH). (**B**–**D**) Quantitative bar chart for each protein’s expression level (mean ± SD, * *p* < 0.05 compared to group treated with 1 µg/mL LPS alone).

To confirm that compound **5** might be correlated with the inhibition of protein expression of IκB kinase alpha and beta (IKKα/β), inhibitor of kappa B alpha (I-κBα), and nuclear factor kappa B (NF-κB), we performed Western blot analysis. When the RAW264.7 cells were stimulated with LPS (1µg/mL) for 24 h, the protein expression of IKKα/β, I-κBα, and NF-κB was increased. However, treatment with compound **5** at 50 µM and 100 µM significantly inhibited expression of IKKα/β, I-κBα, and NF-κB in a concentration dependent manner (Figure 4).

### 2.4. Effects of Compound ***5*** on the LPS-Induced Expression of iNOS and COX-2 in RAW 264.7 Mouse Macrophages

**Figure 5:**
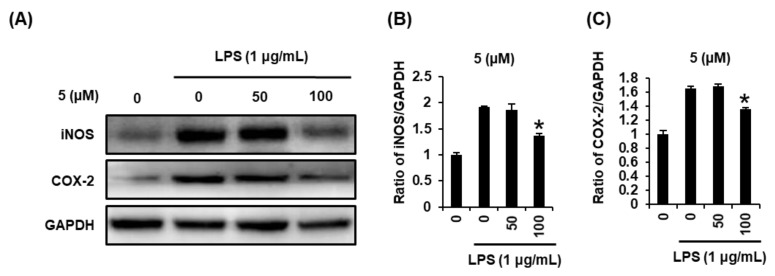
Effects of compound **5** on the expression of inducible nitric oxide synthase (iNOS) and cyclooxygenase-2 (COX-2) in RAW 264.7 mouse macrophages treated with lipopolysaccharide (LPS). (**A**) Representative Western blots showing protein expressions of iNOS, COX-2, and glyceraldehyde-3-phosphate dehydrogenase (GAPDH). (**B**,**C**) Quantitative bar chart for each protein’s expression level (mean ± SD, * *p* < 0.05 compared to group treated with 1 µg/mL LPS alone).

As a result of conducting a follow-up experiment based on the above results, we found that the protein expression of iNOS and COX-2 was increased after stimulation with LPS (1µg/mL) for 24 h, whereas treatment with compound **5** at 50 µM and 100 µM significantly inhibited this overexpression in a concentration dependent manner in RAW264.7 cells (Figure 5).

## 3. Discussion

Many studies have been performed with respect to the anti-inflammatory activity of extract of the mulberry fruit (*Morus alba* L.) [7,34,35,36,37]. In contrast, studies on which compounds in mulberry fruit have anti-inflammatory activity are still lacking.

In our previous study, the anti-inflammatory activity of the ethanol extract of the mulberry fruit was reported [7]. As an extension of previous research, our present study performed phytochemical analysis to isolate 22 chemical constituents from the ethanol extract and their non-toxic concentrations were investigated in RAW 264.7 macrophages. In addition, the inhibitory effect on LPS-stimulated NO production and its underlying molecular mechanism were investigated in RAW 264.7 macrophages. Kang et al. recently reported the inhibitory effect of cyclo(l-Pro-d-Val) on LPS-induced endothelial inflammatory responses [38], but they used different cells from ours, and the related mechanistic studies have not yet been conducted. In the present study, the anti-inflammatory action of cyclo(l-Pro-l-Val) and its mechanism of action were evaluated. These results could be a potential scaffold for the development of therapeutic agents to treat inflammatory disorders.

During the inflammatory response to LPS, RAW 264.7 macrophages play a central role in a regulating overproduction of a pro-inflammatory mediator, NO, in cell-based models of inflammation [39]. In this study, compound **5** markedly inhibited the NO production in a concentration-dependent manner in LPS-treated RAW 264.7 cells. Interestingly, its inhibitory effect was similar to that of L-NMMA. L-NMMA is an inhibitor of NO synthesis [40]. There was a report that evaluated the inhibitory effect of L-NMMA on expressions of IKKα/β, I-κBα, NF-κB, and iNOS in LPS-treated RAW 264.7 cells [41].

In response to LPS, IKK composed of two catalytic subunits (IKKα and IKKβ) are phosphorylated [42]. Phosphorylation of both IKKα and IKKβ leads to IκB phosphorylation, which directly contributes to activation of a nuclear transcription factor, NF-κB [43]. NF-κB regulates the transcription of iNOS and COX-2 by binding to specific DNA sequences. Induction of iNOS and COX-2 produces NO and prostaglandin E2 (PGE2), respectively [44]. Thus, we investigated whether compound **5** could inhibit expressions of IKKα/β, I-κBα, NF-κB, iNOS, and COX-2 in LPS-treated RAW 264.7 cells using Western blot analysis. Our studies demonstrate that compound **5** in a concentration-dependent manner inhibited LPS-mediated overexpressed IKKα/β, I-κBα, and NF-κB in RAW264.7 cells. In addition, increased expression of the iNOS and COX-2 was observed in response to LPS stimulation which was inhibited after treatment with compound **5**. These results indicated that compound **5** inhibited the expression of iNOS and COX-2 via inhibition of NF-κB/I-κBα pathway, thus lower expression of iNOS resulted in lower NO production (Figure 5). Although more experiments including animal research are needed to clarify effect bioavailability and bio-accessibility, compound **5** has potential as a favorable candidate for the treatment of inflammatory diseases.

## 4. Materials and Methods

### 4.1. General Experimental Procedures

Optical rotations were measured by a Jasco P-1020 polarimeter (Jasco, Easton, MD, USA). IR spectra were recorded by a Bruker IFS-66/S FT-IR spectrometer (Bruker, Karlsruhe, Germany). Electrospray ionization (ESI) mass spectra were recorded on an Agilent 1200 Series HPLC system (Agilent Technologies, Santa Clara, CA, USA), equipped with a diode array detector and 6130 Series ESI mass spectrometer using an analytical Kinetex C_18_ 100 Å column (100 × 2.1 mm, 5 µm; Phenomenex, Torrance, CA, USA; solvent condition: from 10% MeOH/H_2_O to 100% MeOH (gradient system, 0–20 min); flow rate: 0.3 mL/min). Nuclear magnetic resonance (NMR) spectra were recorded by a Bruker AVANCE III 700 NMR spectrometer operating at 700 MHz (^1^H) and 175 MHz (^13^C) (Bruker, Karlsruhe, Germany), with chemical shifts given in ppm (δ). The ^1^H NMR spectrum of compound **5** was recorded using a Bruker AVANCE III HD 850 NMR spectrometer with a 5 mm TCI CryoProbe operating at 850 MHz, with chemical shifts given in ppm (*δ*). Preparative high-performance liquid chromatography (HPLC) used a Waters 1525 Binary HPLC pump with Waters 996 Photodiode Array Detector (Waters Corporation, Milford, CT, USA). Semi-preparative HPLC used a Shimadzu Prominence HPLC System with SPD-20A/20AV Series Prominence HPLC UV-Vis Detectors (Shimadzu, Tokyo, Japan) and a Phenomenex Luna C18 column (250 × 10 mm, 5 µm; flow rate: 2 mL/min; Phenomenex, Torrance, CA, USA). Silica gel 60 (Merck, 70–230 mesh and 230–400 mesh) and RP-C18 silica gel (Merck, 40–63 µm) were used for column chromatography. Merck precoated Silica gel F254 plates and RP-18 F254s plates (Merck, Darmstadt, Germany) were used for TLC. Spots were detected on TLC under UV light or by heating after spraying with anisaldehyde-sulfuric acid.

### 4.2. Plant Material

The fruits of *M. alba* were collected in China in January 2014. A voucher specimen (MA 1414) of the material was identified by one of the authors (K.H. Kim) and was placed in the herbarium of the School of Pharmacy, Sungkyunkwan University, Suwon, Korea.

### 4.3. Extraction and Isolation

*M. alba* fruits (10.5 kg) were dried at room temperature for one week and pulverized, and then immersed with a 70% ethanol (2.5 L) for two days three times at room temperature. The resultant ethanol extract was evaporated in vacuo, affording a crude brown ethanol extract (1.4 kg). The extract was dissolved in distilled water (700 mL) solvent-partitioned using hexane, CH_2_Cl_2_, EtOAc, and BuOH to obtain the major four fractions, yielding 27.8, 85.3, 32.9, and 138.8 g, respectively. The CH_2_Cl_2_ fraction was loaded onto a silica gel column (230–400 mesh) column and fractionated with CH_2_Cl_2_–MeOH (50:1–1:1, gradient system) to yield five fractions (A1–A5). Fraction A2 (2.3 g) was separated utilizing RP-C18 silica gel (230–400 mesh) column chromatography with 70% aqueous MeOH to give eleven fractions (B1–B11). Fraction B2 (753 mg) was subjected to silica gel column (230–400 mesh) eluted with CH_2_Cl_2_–MeOH (50:1–1:1, gradient system) to afford eight subfractions (B21–B28). Compounds **4** (2.0 mg, t_R_ = 22.0 min, ESIMS (positive-ion mode) m/z 116.0 [M + H]^+^), **5** (1.8 mg, t_R_ = 30.0 min, ESIMS (positive-ion mode) m/z 197.1 [M + H]^+^), and **6** (2.9 mg, t_R_ = 45.5 min, ESIMS (positive-ion mode) m/z 137.0 [M + H]^+^) were purified from subfraction B22 (84 mg) by semi-preparative reversed-phase HPLC using 35% aqueous MeOH. Subfraction B23 (86 mg) was separated by semi-preparative reversed-phase HPLC using 40% aqueous MeOH to purify compounds **9** (1.5 mg, t_R_ = 45.5 min, ESIMS (positive-ion mode) m/z 139.0 [M + H]^+^) and **10** (3.5 mg, t_R_ = 50.5 min, ESIMS (positive-ion mode) m/z 167.0 [M + H]^+^). Subfraction B24 (108 mg) was fractionated by preparative reversed-phase HPLC eluted with MeOH-H_2_O (1:9–1:0, gradient system) to obtain five subfractions (B241–B245). Compounds **7** (2.4 mg, t_R_ = 15.0 min, ESIMS (positive-ion mode) m/z 171.1 [M + H]^+^) and **8** (2.9 mg, t_R_ = 32.5 min, ESIMS (positive-ion mode) m/z 169.0 [M + H]^+^) were isolated from subfraction B242 (10 mg) by semi-preparative reversed-phase HPLC using 52% aqueous MeOH. Compounds **1** (3.0 mg, t_R_ = 37.0 min, ESIMS (positive-ion mode) m/z 225.1 [M + H]^+^), **2** (4.2 mg, t_R_ = 40.0 min, ESIMS (positive-ion mode) m/z 183.1 [M + H]^+^), and **3** (2.8 mg, t_R_ = 45.0 min, ESIMS (positive-ion mode) m/z 379.1 [M + H]^+^) were obtained from subfraction B243 (14 mg) by semi-preparative reversed-phase HPLC using 59% aqueous MeOH. Fraction B8 (445 mg) was fractionated by silica gel column (230–400 mesh) with CH_2_Cl_2_–MeOH (30:1–1:1, gradient system) to afford nine subfractions (B81–B88). Compounds **11** (3.8 mg, t_R_ = 30.4 min, ESIMS (positive-ion mode) m/z 283.2 [M + H]^+^) and **12** (5.5 mg, t_R_ = 48.0 min, ESIMS (positive-ion mode) m/z 431.3 [M + H]^+^) were isolated from subfraction B83 (38 mg) utilizing semi-preparative reversed-phase HPLC using 89% aqueous MeOH. Four subfractions (B91–B94) were obtained from subfraction B9 (398 mg) using a silica gel column (230–400 mesh) with CH_2_Cl_2_–MeOH (50:1–1:1, gradient system). Subfraction B91 (25 mg) was separated by semi-preparative reversed-phase HPLC using 91% aqueous MeOH to obtain compounds **13** (6.0 mg, t_R_ = 42.0 min, ESIMS (positive-ion mode) m/z 429.3 [M + H]^+^) and **14** (7.2 mg, t_R_ = 47.0 min, ESIMS (positive-ion mode) m/z 429.3 [M + H]^+^). Subfraction B93 (38 mg) was separated utilizing semi-preparative reversed-phase HPLC eluted with 92% aqueous MeOH to obtain compounds **15** (4.0 mg, t_R_ = 51.5 min, ESIMS (positive-ion mode) m/z 431.3 [M + H]^+^), **16** (6.7 mg, t_R_ = 53.0 min, ESIMS (positive-ion mode) m/z 431.3 [M + H]^+^), and **22** (2.8 mg, t_R_ = 54.0 min, ESIMS (positive-ion mode) m/z 445.3 [M + H]^+^). Fraction B10 (315 mg) was fractionated by a silica gel column (230–400 mesh) eluted with CH_2_Cl_2_–MeOH (100:1–1:1, gradient system) to give four subfractions (B101-B104). Compounds **17** (3.6 mg, t_R_ = 43.0 min, ESIMS (positive-ion mode) m/z 445.4 [M + H]^+^) and **18** (4.0 mg, t_R_ = 43.5 min, ESIMS (positive-ion mode) m/z 445.4 [M + H]^+^) were purified from subfraction B101 (23 mg) utilizing semi-preparative reversed-phase HPLC eluted with 88% aqueous MeOH. Fraction A3 (1.8 g) was fractionated by a silica gel column (230–400 mesh) eluted with CH_2_Cl_2_–MeOH (100:1–1:1, gradient system) to acquire seven subfractions (A31–A37). Fraction A37 (330 mg) was further fractionated by a silica gel column (230–400 mesh) eluted with CH_2_Cl_2_–MeOH (30:1–1:1, gradient system) to give three subfractions (A371–373). Compounds **19** (6.3 mg, t_R_ = 32.5 min, ESIMS (positive-ion mode) m/z 593.4 [M + H]^+^), **20** (4.2 mg, t_R_ = 51.0 min, ESIMS (positive-ion mode) m/z 702.5 [M + H]^+^), and **21** (3.1 mg, t_R_ = 52.5 min, ESIMS (positive-ion mode) m/z 593.4 [M + H]^+^) were purified from subfraction A372 (139 mg) using semi-preparative reversed-phase HPLC eluted with 85% aqueous MeOH.

### 4.4. RAW 264.7 Cells Culture

A mouse macrophage cell line, RAW 264.7 (American Type Culture Collection, Rockville, MD, USA), was cultured in DMEM (Manassas, VA, USA) containing 4 mM L-glutamine, antibiotics (1% penicillin/streptomycin), and 10% fetal bovine serum in humidified air environment at 37 °C in a 5% CO_2_.

### 4.5. Measurement of Viability of RAW 264.7 Cells

RAW 264.7 cells (3 × 10^4^ cells/well) were exposed to the indicated concentrations of compounds **1**–**22** for 24 h at 37 °C and incubated for an additional 40 min with Ez-Cytox solution (Daeil Lab Service Co., Seoul, Korea). Optical density at 450 nm was determined using a spectrophotometer microplate (PowerWave XS; Bio-Tek Instruments, Winooski, VT, USA).

### 4.6. Measurement of NO Produced by RAW 264.7 Cells

RAW 264.7 cells (3 × 10^4^ cells/well) were exposed to the indicated concentrations of compounds **1**–**22** for 1 h and then incubated for an additional 24 h with LPS (1 µg/mL). At the end of the incubation, each culture supernatant was blended with the Griess reagent to determine NO production by RAW 264.7 cells. Optical density at 540 nm of the mixture was determined using a spectrophotometer microplate (PowerWave XS; Bio-Tek Instruments, Winooski, VT, USA).

### 4.7. Western Blot Analysis

RAW 264.7 cells (4 × 10^5^ cells/well) were exposed to the indicated concentrations of compound **5** for 1 h, and then incubated for an additional 24 h with LPS (1 µg/mL). At the end of the incubation, the RAW 264.7 cells were lysed with lysis buffer (Cell Signaling Technology, Beverly, MA, USA), supplemented with 1 mM phenylmethylsulfonyl fluoride, for 20 min. For Western blot analysis, 20 µg of the total protein from the cell lysate was separated by 10% sodium dodecyl sulfate–polyacrylamide gel electrophoresis (SDS-PAGE). The proteins were electro-transferred to a polyvinylidene fluoride (PVDF) membrane. Each PVDF membrane was probed with primary antibodies (Cell Signaling Technology, Beverly, MA, USA) overnight, then incubated with horse radish peroxidase-conjugated anti-rabbit antibodies (Cell Signaling Technology, Beverly, MA, USA) for 1 h at room temperature, and visualized using an enhanced chemiluminescence detection reagent (GE Healthcare, Little Chalfont, UK). Western blot signals were detected by FUSION Solo Chemiluminescence System (PEQLAB Biotechnologie GmbH, Erlangen, Germany).

### 4.8. Statistical Analysis

All assays were performed in triplicate and repeated at least three times. All data are presented as the mean ± standard deviation (SD). Statistical significance was determined using one-way analysis of variance (ANOVA) and multiple comparisons with the Bonferroni correction. A *p* value of <0.05 indicated statistical significance. All analyses were performed using SPSS Statistics ver. 19.0 (SPSS Inc., Chicago, IL, USA).

## 5. Conclusions

**Figure 6:**
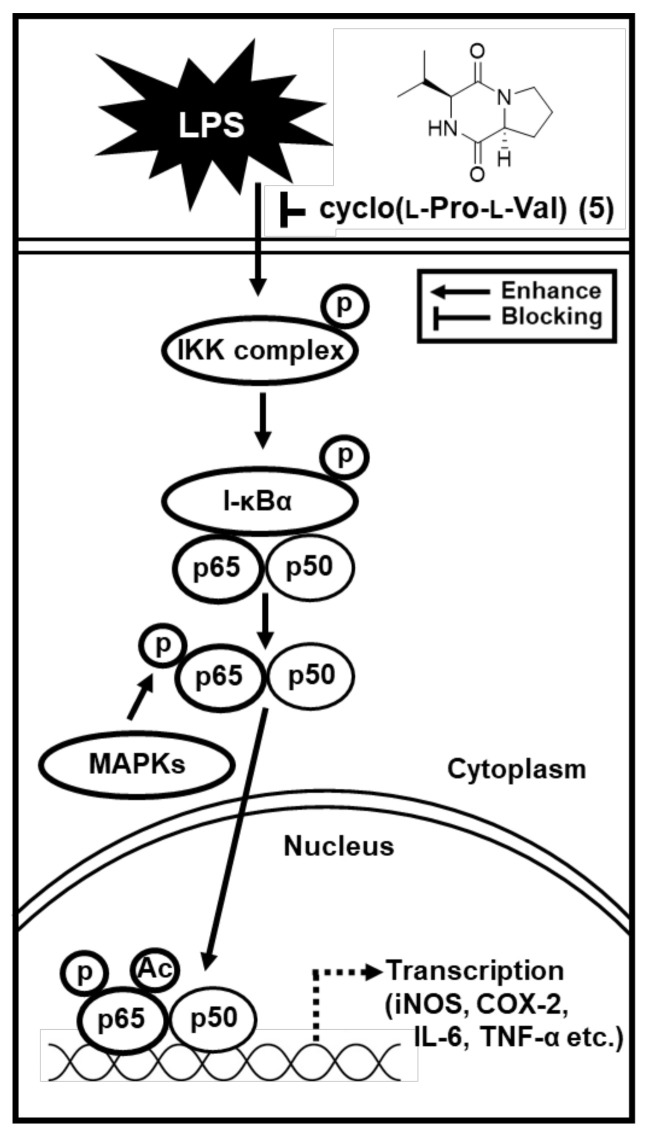
Schematic illustration of the mechanism for the potential role of compound **5** isolated from *M. alba* fruits in inflammatory responses. LPS, lipopolysaccharide; p, phosphorylated; IKK, IκB kinase alpha; IκBα, inhibitor of kappa B alpha; p56 and p50, cellular proteins; MAPK, mitogen-activated protein kinase; Ac, activated; TNF-α, tumor necrosis factor alpha; IL-6, interleukin 6; COX-2, cyclooxygenase-2; iNOS, inducible nitric oxide synthase.

In summary, as part of an ongoing research project to discover bioactive natural products [45,46,47,48,49,50], phytochemical examination of the extract of *M. alba* fruits led to the isolation and identification of 22 compounds in the process of discovery of potential anti-inflammatory compounds. This study demonstrates that compound **5** inhibited NO production and iNOS and COX-2 in LPS-stimulated RAW 264.7 macrophages. At least in part, these inhibitory effects are mediated via inhibition of NF-κB/I-κBα pathway (Figure 6). Thus, these findings supported the utilization of cyclo(L-Pro-L-Val) (**5**) as a favorable candidate for the treatment of inflammatory diseases.

## Data Availability

The data that support the findings of this study are available from the corresponding author upon reasonable request.

## Supplementary Materials

The following are available online at https://www.mdpi.com/article/10.3390/ijms22158120/s1, Figure S1: ^1^H-NMR spectrum of compound **1** (in CD_3_OD, 700 MHz); Figure S2: ^1^H-NMR spectrum of compound **2** (in CD_3_OD, 700 MHz); Figure S3: ^1^H-NMR spectrum of compound **3** (in CD_3_OD, 700 MHz); Figure S4: ^1^H-NMR spectrum of compound **4** (in CD_3_OD, 700 MHz); Figure S5: ^13^C-NMR spectrum of compound **4** (in CD_3_OD, 175 MHz); Figure S6: ^1^H-NMR spectrum of compound **5** (in CD_3_OD, 700 MHz); Figure S7: ^1^H-NMR spectrum of compound **5** (in CDCl_3_, 850 MHz); Figure S8: ^1^H-NMR spectrum of compound **6** (in CD_3_OD, 700 MHz); Figure S9: ^1^H-NMR spectrum of compound **7** (in CD_3_OD, 700 MHz); Figure S10: ^1^H-NMR spectrum of compound **8** (in CD_3_OD, 700 MHz); Figure S11: ^1^H-NMR spectrum of compound **9** (in CD_3_OD, 700 MHz); Figure S12: ^1^H-NMR spectrum of compound **10** (in CD_3_OD, 700 MHz); Figure S13: ^1^H-NMR spectrum of compound **11** (in CD_3_OD, 700 MHz); Figure S14: ^1^H-NMR spectrum of compound **12** (in CDCl_3,_ 700 MHz); Figure S15: ^1^H-NMR spectrum of compound **13** (in CDCl_3,_ 700 MHz); Figure S16: ^1^H-NMR spectrum of compound **14** (in CDCl_3,_ 700 MHz); Figure S17: ^1^H-NMR spectrum of compound **15** (in CD_3_OD, 700 MHz); Figure S18: ^1^H-NMR spectrum of compound **16** (in CDCl_3,_ 700 MHz); Figure S19: ^1^H-NMR spectrum of compound **17** (in CD_3_OD, 700 MHz); Figure S20: ^1^H-NMR spectrum of compound **18** (in CD_3_OD, 700 MHz); Figure S21: ^1^H-NMR spectrum of compound **19** (in CD_3_OD, 700 MHz); Figure S22: ^1^H-NMR spectrum of compound **20** (in CD_3_OD, 700 MHz); Figure S23: ^1^H-NMR spectrum of compound **21** (in CD_3_OD, 700 MHz); Figure S24: ^1^H-NMR spectrum of compound **22** (in CD_3_OD, 700 MHz); Figure S25: The retention time of the L-FDAA derivatized L-Val as a standard; Figure S26: The retention time of the L-FDAA derivatized D-Val as a standard; Figure S27: The retention time of the L-FDAA derivatized L-Pro as a standard; Figure S28: The retention time of the L-FDAA derivatized D-Pro as a standard; Figure S29: The retention times of the L-FDAA derivatized Val/Pro derived from compound **5**; Figure S30: The retention times of the L-FDAA derivatized Val derived from compound **5**; Figure S31: The retention times of the L-FDAA derivatized Pro derived from compound **5**.
