# Supplementary material for: Bioactive Phytochemicals from Mulberry: Potential Anti-Inflammatory Effects in Lipopolysaccharide-Stimulated RAW 264.7 Macrophages
Source: Int J Mol Sci. 2021 Jul 29;22(15):8120. doi: 10.3390/ijms22158120 (PMC8348635; doi:10.3390/ijms22158120)
Supplement: Supplementary file 1 [file ijms-22-08120-s001.zip › ijms-1261224-supplementary.pdf]

Supplementary Materials

Bioactive Phytochemicals from Mulberry: Potential Anti-inflammatory Effects in Lipopolysaccharide-stimulated RAW 264.7 Macrophages

Dahae Lee [1,†], Seoung Rak Lee [2,†], Ki Sung Kang [1,*], and Ki Hyun Kim [2,*]

[1] College of Korean Medicine, Gachon University, Seongnam 13120, Republic of Korea; pjsldh@gachon.ac.kr (D.L.)

[2] School of Pharmacy, Sungkyunkwan University, Suwon 16419, Republic of Korea; davidseoungrak@gmail.com (S.R.L.)

* Correspondence: kkang@gachon.ac.kr (K.S.K.); khkim83@skku.edu (K.H.K.); Tel.: +82-31-750-5402 (K.S.K.); +82-31-290-7700 (K.H.K.)

† These authors contributed equally to this study.

Figure S1: ^{1}H-NMR spectrum of compound **1** (in CD_3OD, 700 MHz)

Figure S2: ^{1}H-NMR spectrum of compound **2** (in CD_3OD, 700 MHz)

Figure S3: ^{1}H-NMR spectrum of compound **3** (in CD_3OD, 700 MHz)

Figure S4: ^{1}H-NMR spectrum of compound **4** (in CD_3OD, 700 MHz)

Figure S5: ^{13}C-NMR spectrum of compound **4** (in CD_3OD, 175 MHz)

Figure S6: ^{1}H-NMR spectrum of compound **5** (in CD_3OD, 700 MHz)

Figure S7: ^{1}H-NMR spectrum of compound **6** (in CD_3OD, 700 MHz)

Figure S8: ^{1}H-NMR spectrum of compound **7** (in CD_3OD, 700 MHz)

Figure S9: ^{1}H-NMR spectrum of compound **8** (in CD_3OD, 700 MHz)

Figure S10 : ^{1}H-NMR spectrum of compound **9** (in CD_3OD, 700 MHz)

Figure S11: ^{1}H-NMR spectrum of compound **10** (in CD_3OD, 700 MHz)

Figure S12: ^{1}H-NMR spectrum of compound **11** (in CD_3OD, 700 MHz)

Figure S13: ^{1}H-NMR spectrum of compound **12** (in $CDCl_3$, 700 MHz)

Figure S14: ^{1}H-NMR spectrum of compound **13** (in $CDCl_3$, 700 MHz)

Figure S15: ^{1}H-NMR spectrum of compound **14** (in $CDCl_3$, 700 MHz)

Figure S16 : ^{1}H-NMR spectrum of compound **15** (in CD_3OD, 700 MHz)

Figure S17: ^{1}H-NMR spectrum of compound **16** (in $CDCl_3$, 700 MHz)

Figure S18: ^{1}H-NMR spectrum of compound **17** (in CD_3OD, 700 MHz)

Figure S19: ^{1}H-NMR spectrum of compound **18** (in CD_3OD, 700 MHz)

Figure S20: ^{1}H-NMR spectrum of compound **19** (in CD_3OD, 700 MHz)

Figure S21: ^{1}H-NMR spectrum of compound **20** (in CD_3OD, 700 MHz)

Figure S22: ^{1}H-NMR spectrum of compound **21** (in CD_3OD, 700 MHz)

Figure S23: ^{1}H-NMR spectrum of compound **22** (in CD_3OD, 700 MHz)

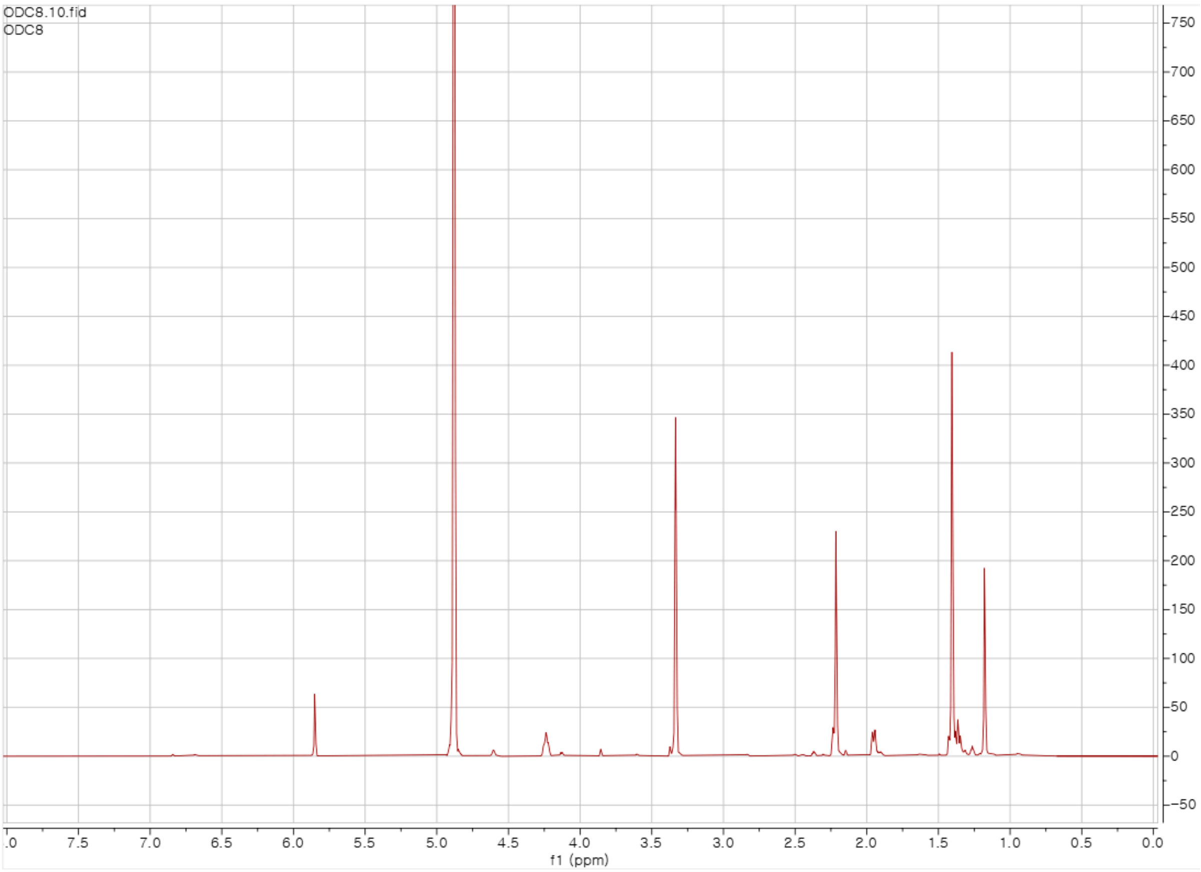

Figure S1. ^{1}H-NMR spectrum of compound **1** (in CD_3OD, 700 MHz)

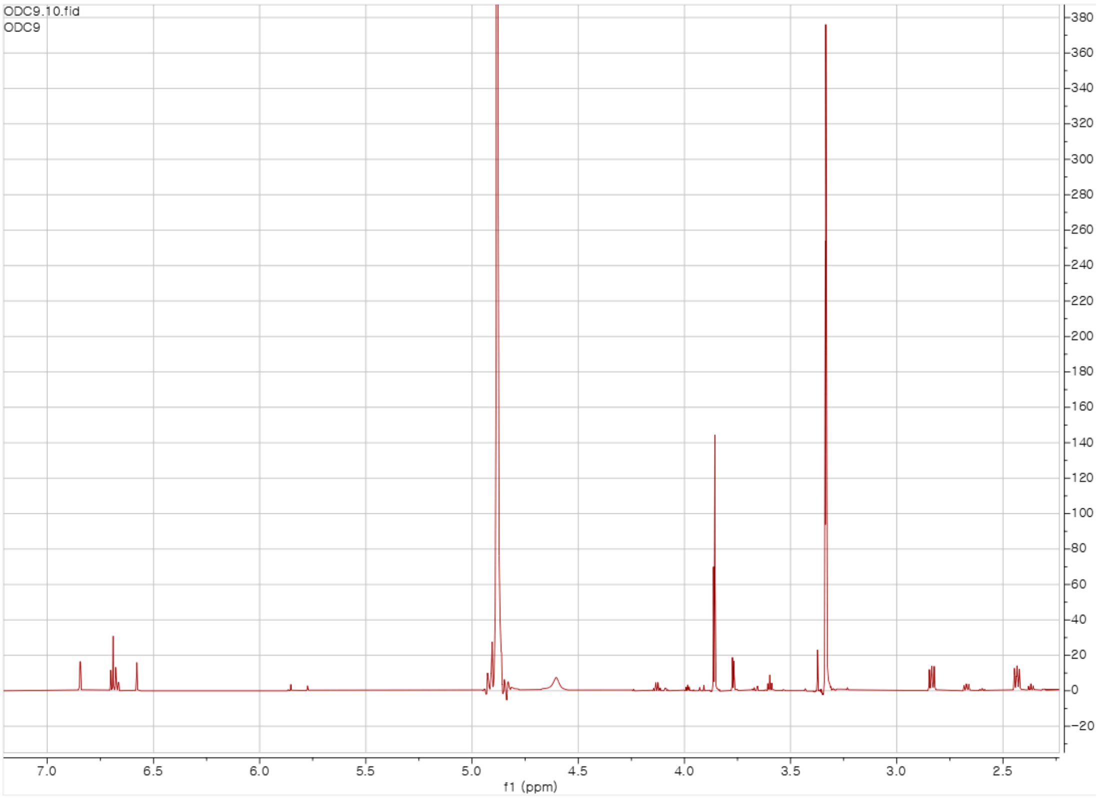

Figure S2. ^{1}H-NMR spectrum of compound **2** (in CD_3OD, 700 MHz)

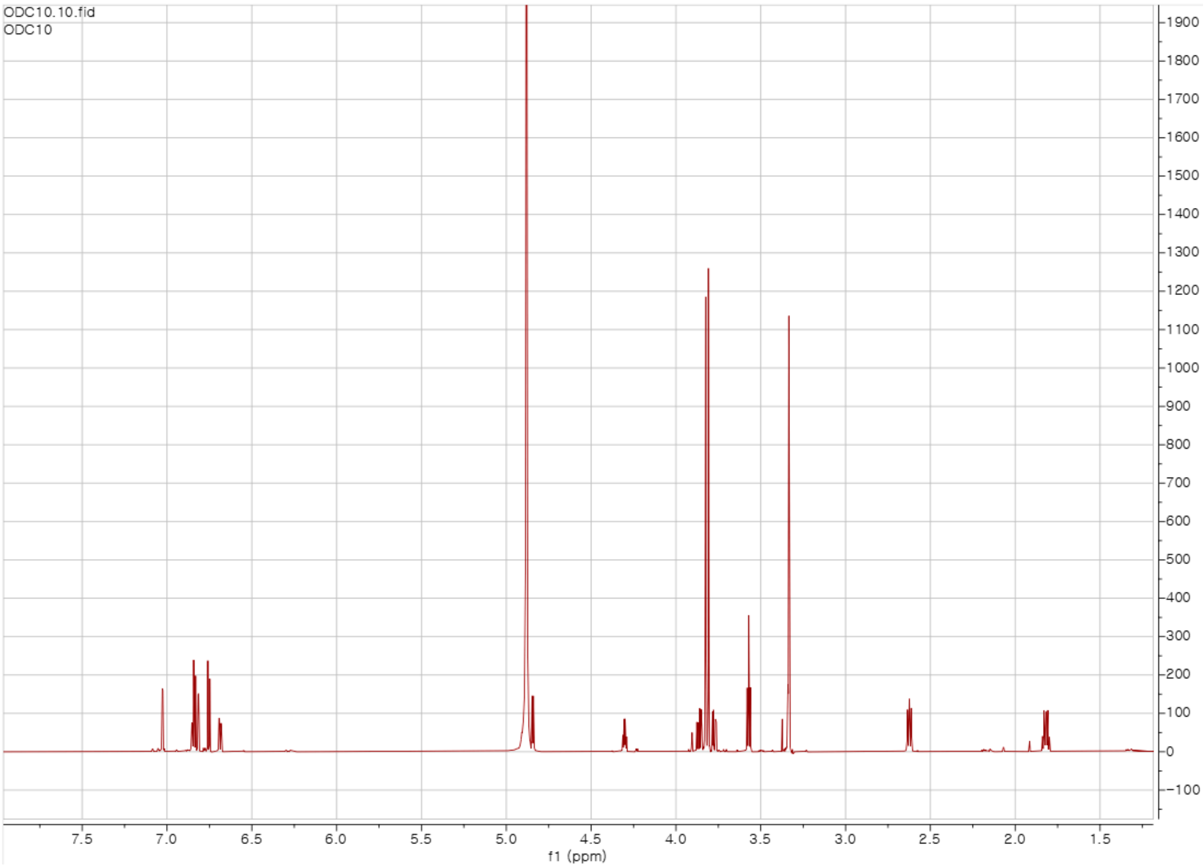

Figure S3. ^{1}H-NMR spectrum of compound **3** (in CD_3OD, 700 MHz)

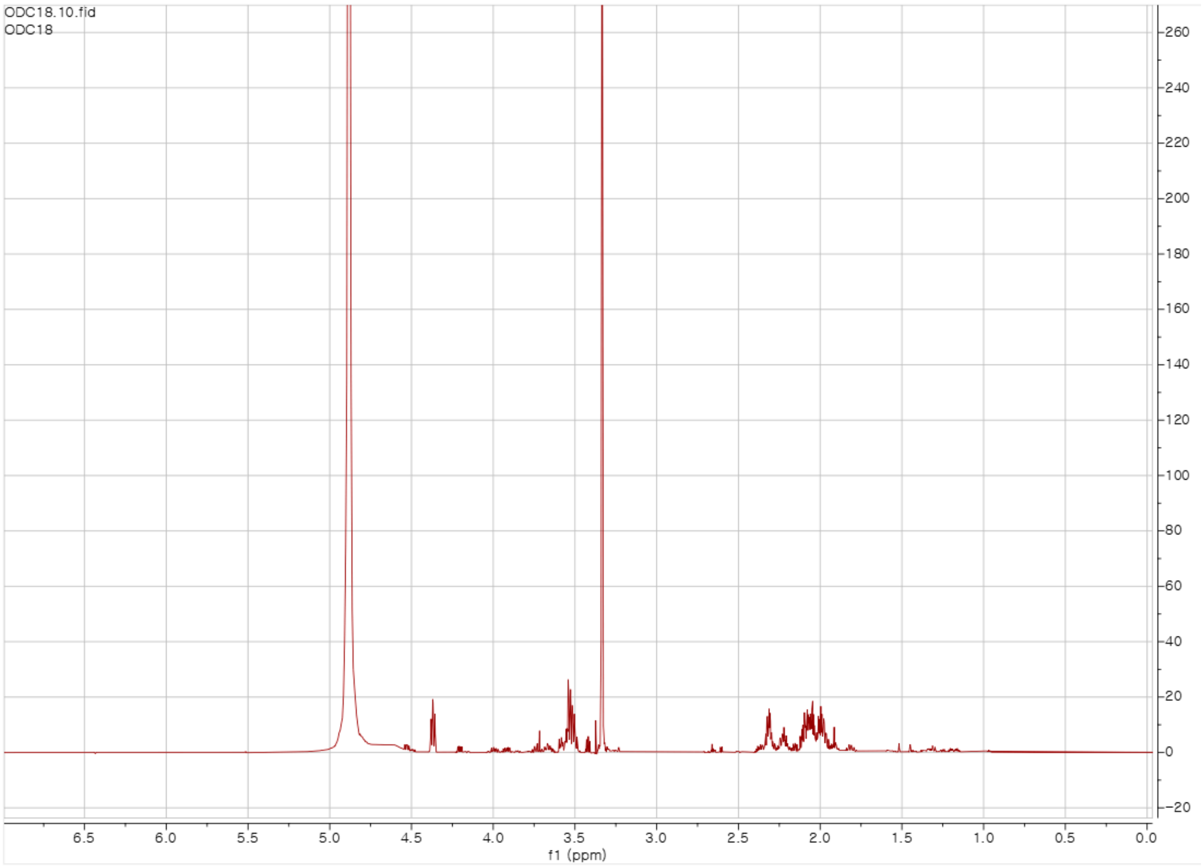

Figure S4. ^{1}H-NMR spectrum of compound **4** (in CD_3OD, 700 MHz)

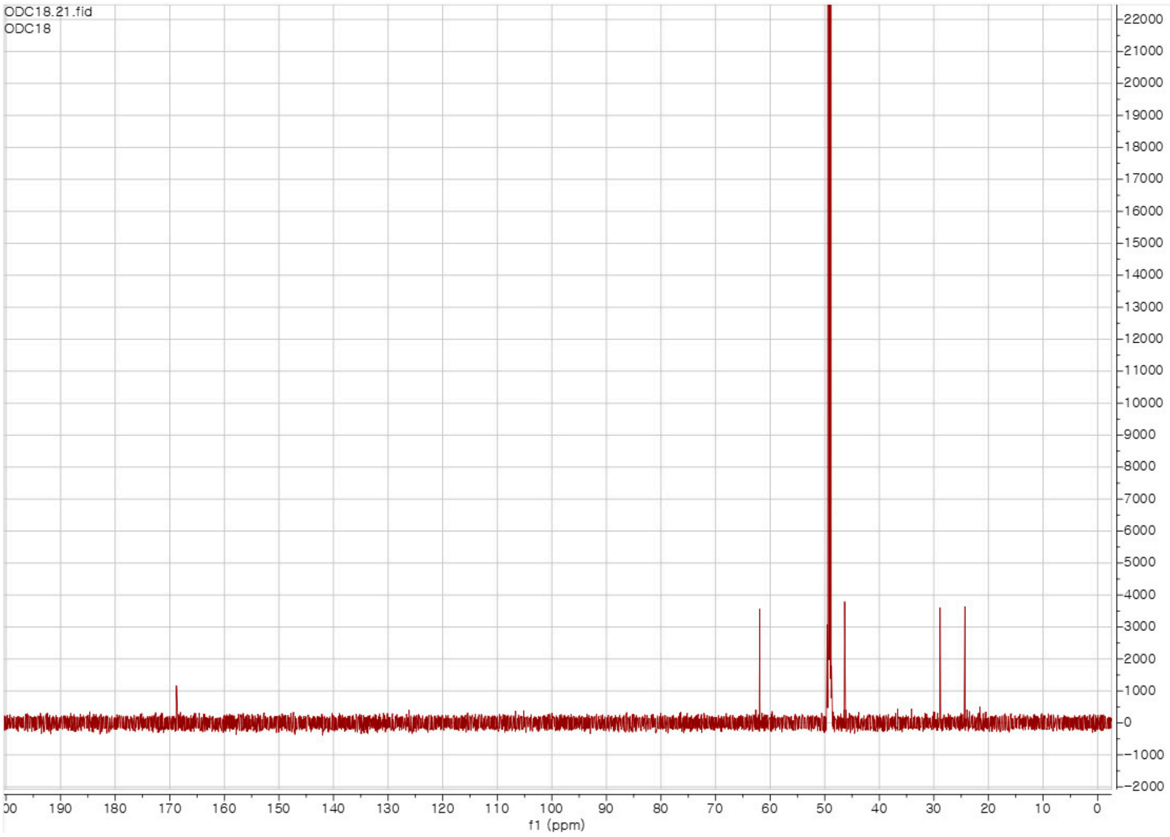

Figure S5. ^{13}C-NMR spectrum of compound **4** (in CD_3OD, 175 MHz)

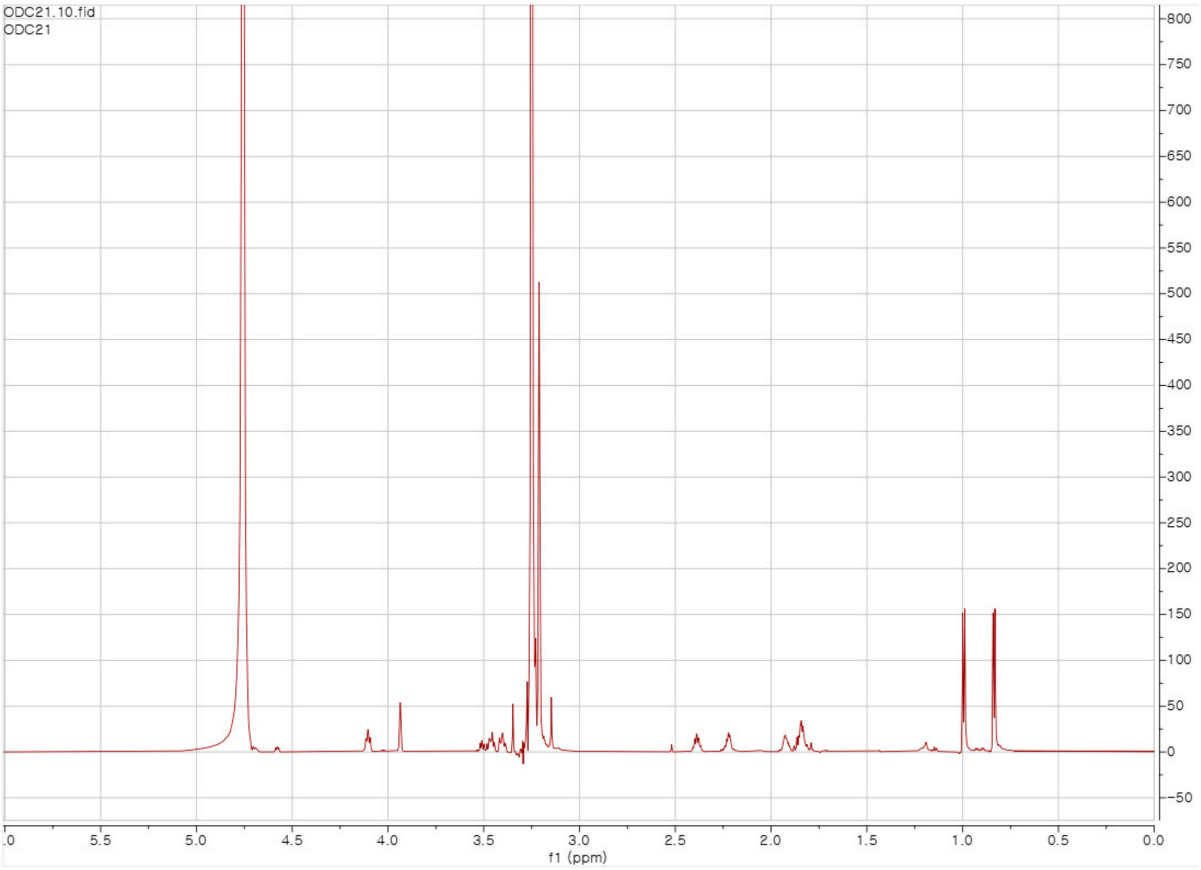

Figure S6. ^{1}H-NMR spectrum of compound **5** (in CD_3OD, 700 MHz)

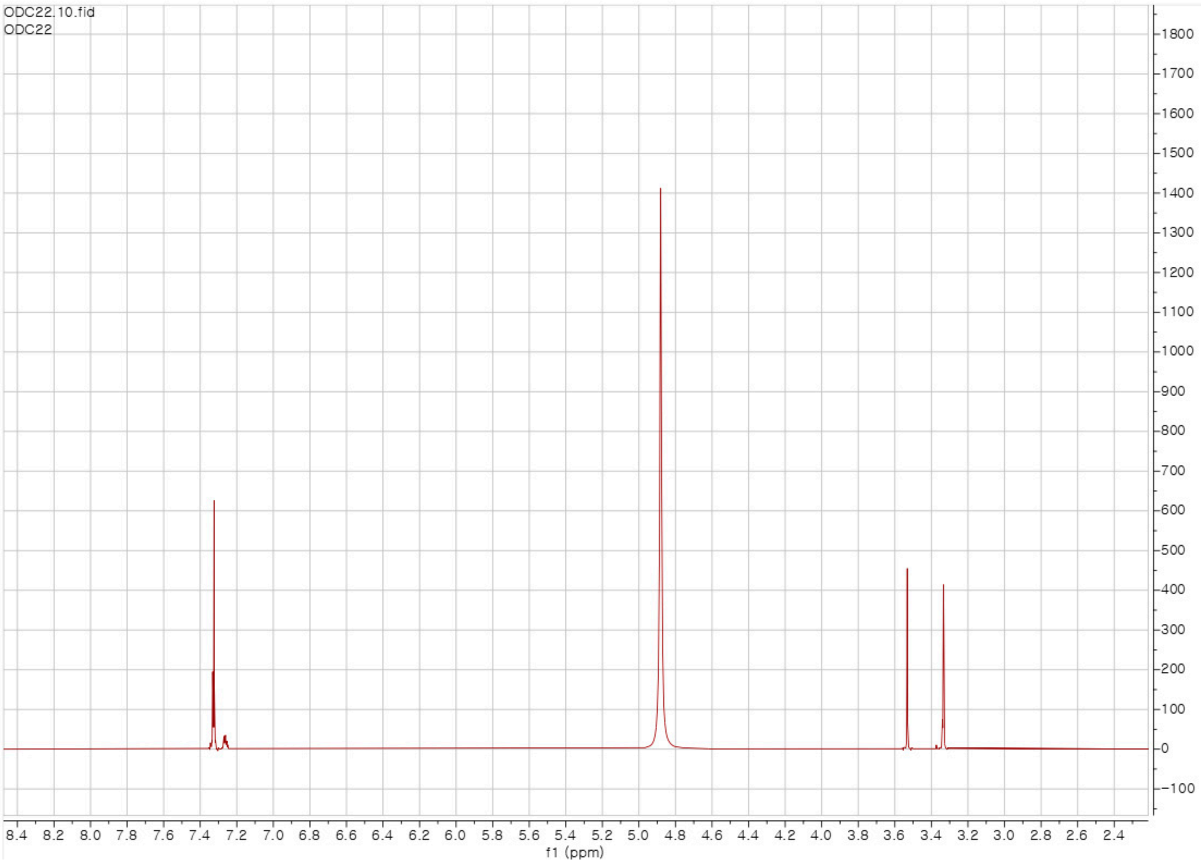

Figure S7. ^{1}H-NMR spectrum of compound **6** (in CD_3OD, 700 MHz)

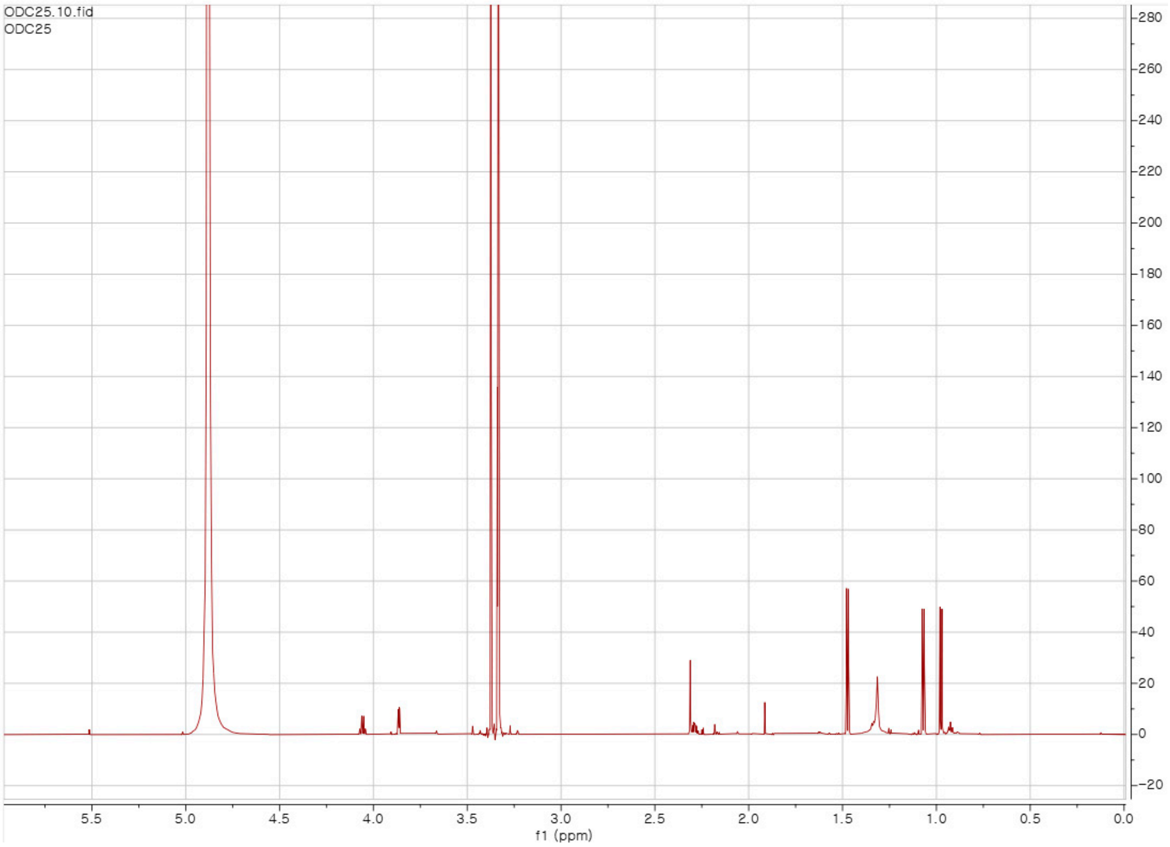

Figure S8. ^{1}H-NMR spectrum of compound **7** (in CD_3OD, 700 MHz)

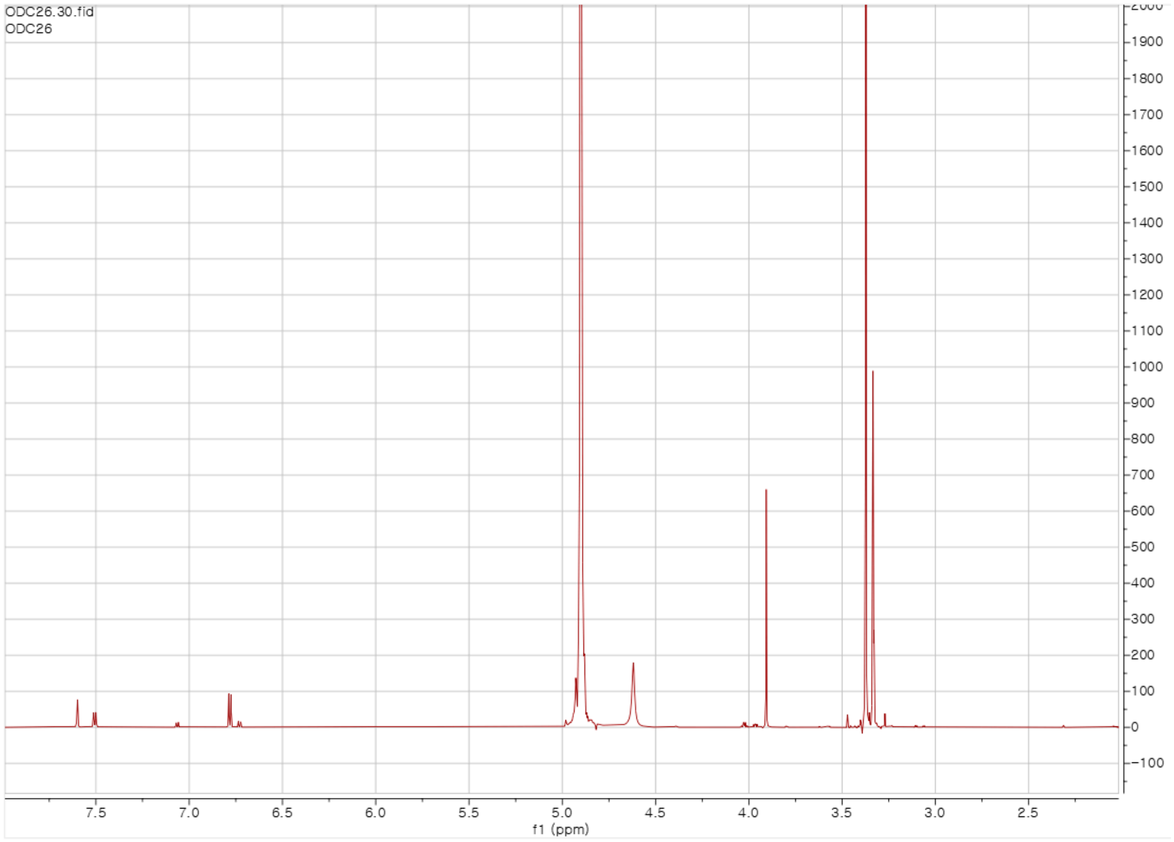

Figure S9. ^{1}H-NMR spectrum of compound **8** (in CD_3OD, 700 MHz)

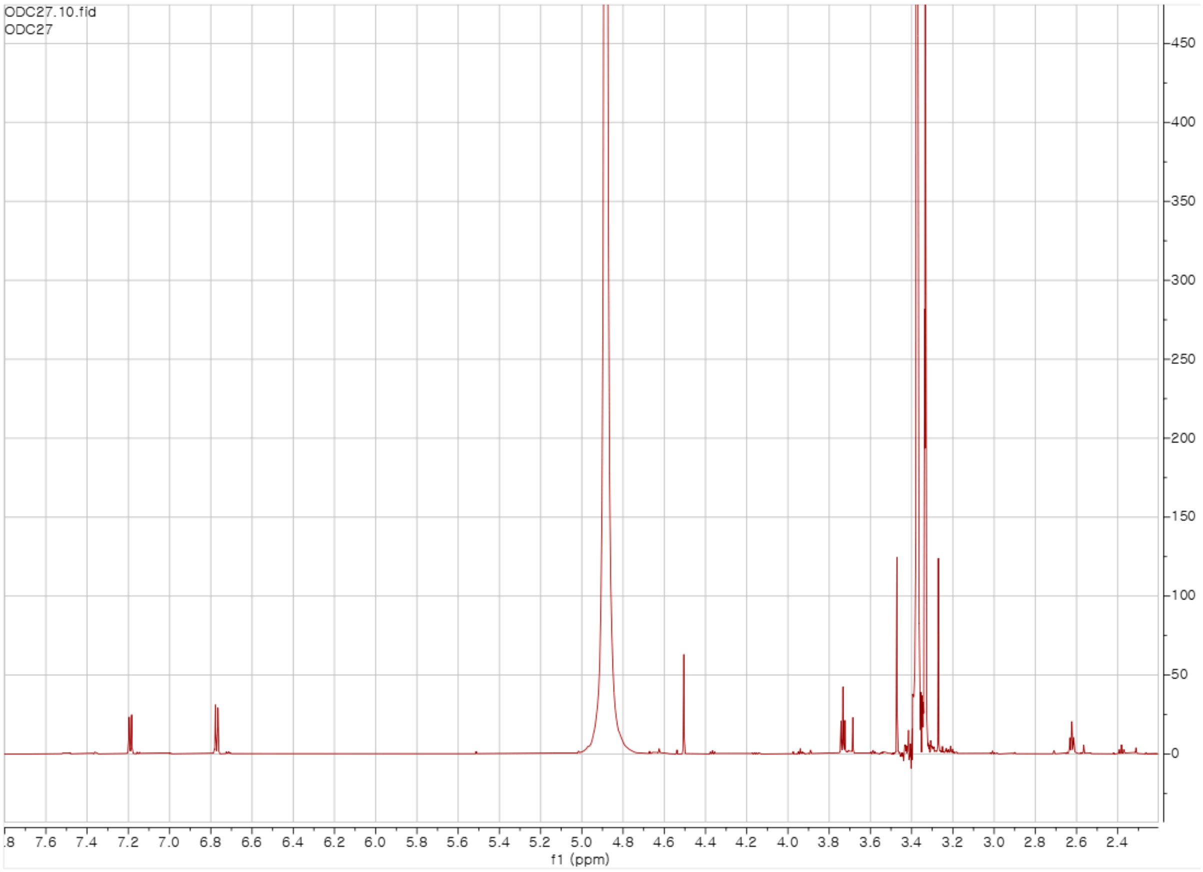

Figure S10. ^{1}H-NMR spectrum of compound **9** (in CD_3OD, 700 MHz)

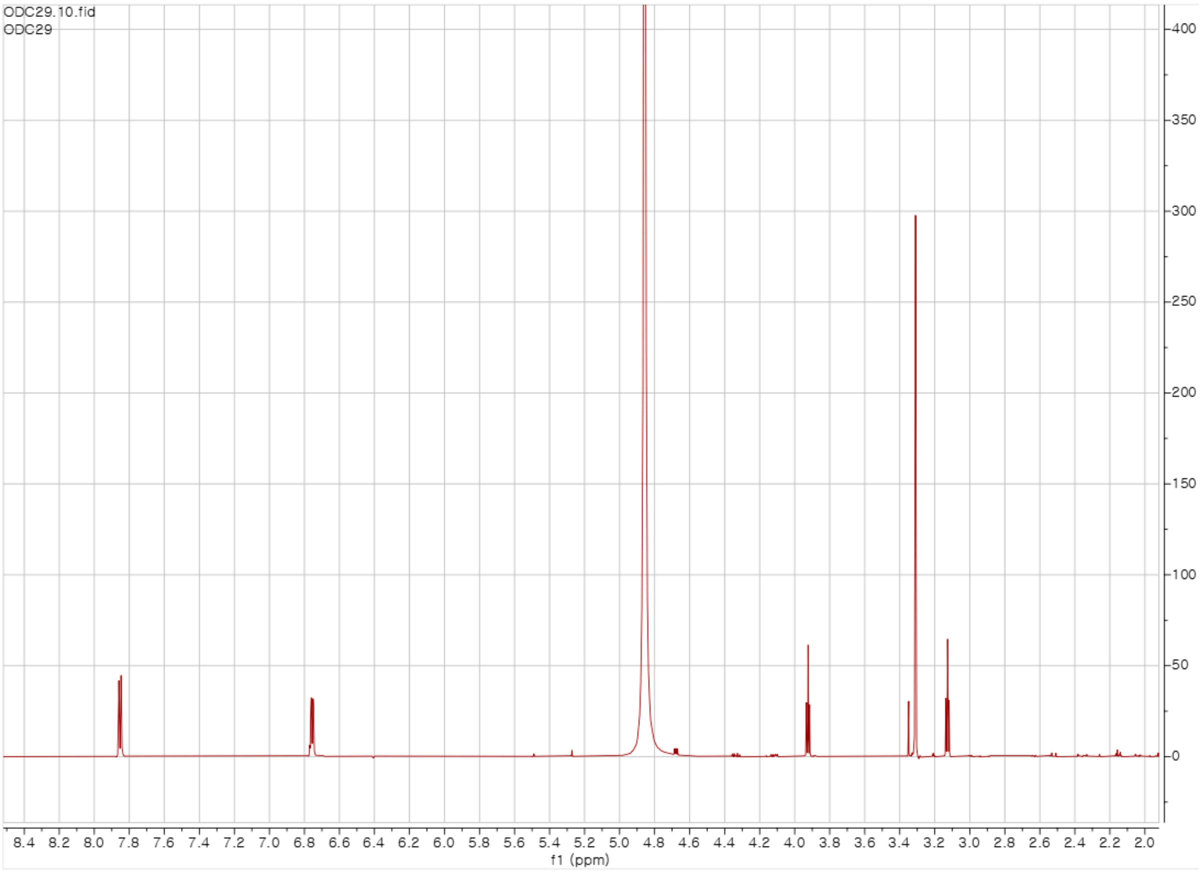

Figure S11. ^{1}H-NMR spectrum of compound **10** (in CD_3OD, 700 MHz)

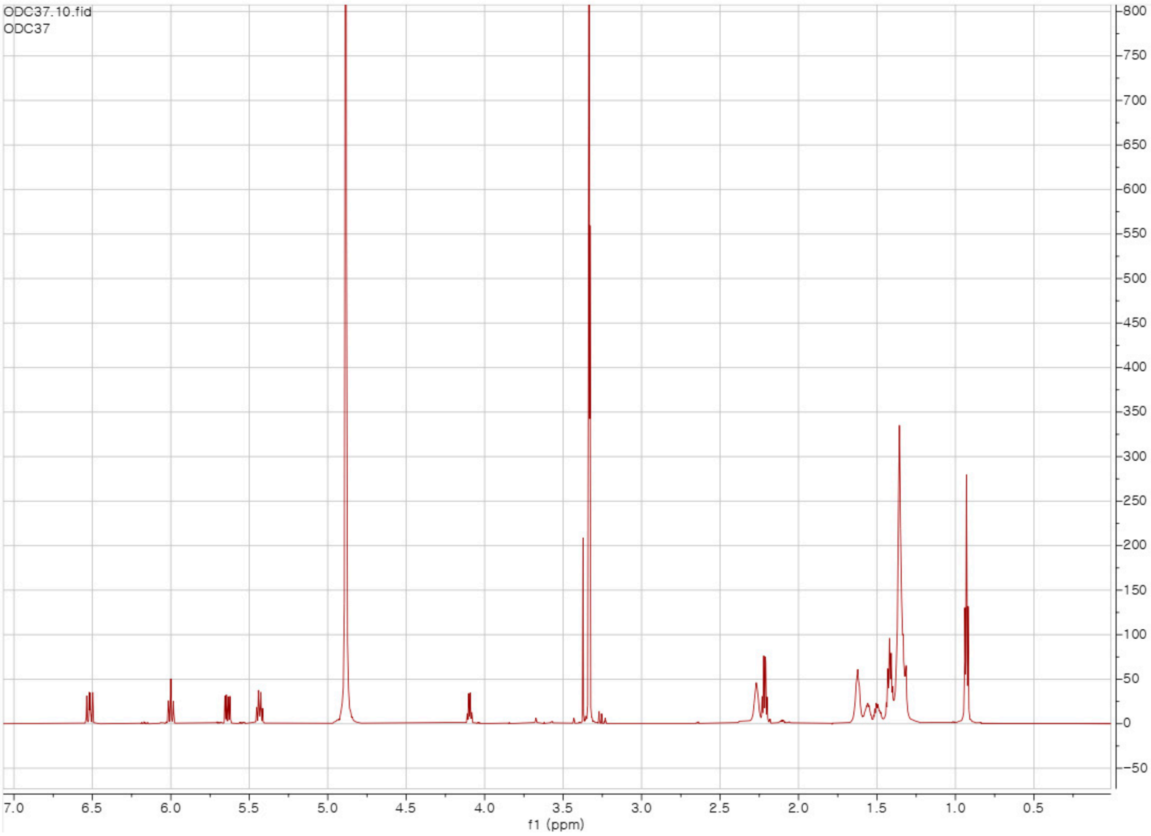

Figure S12. ^{1}H-NMR spectrum of compound **11** (in CD_3OD, 700 MHz)

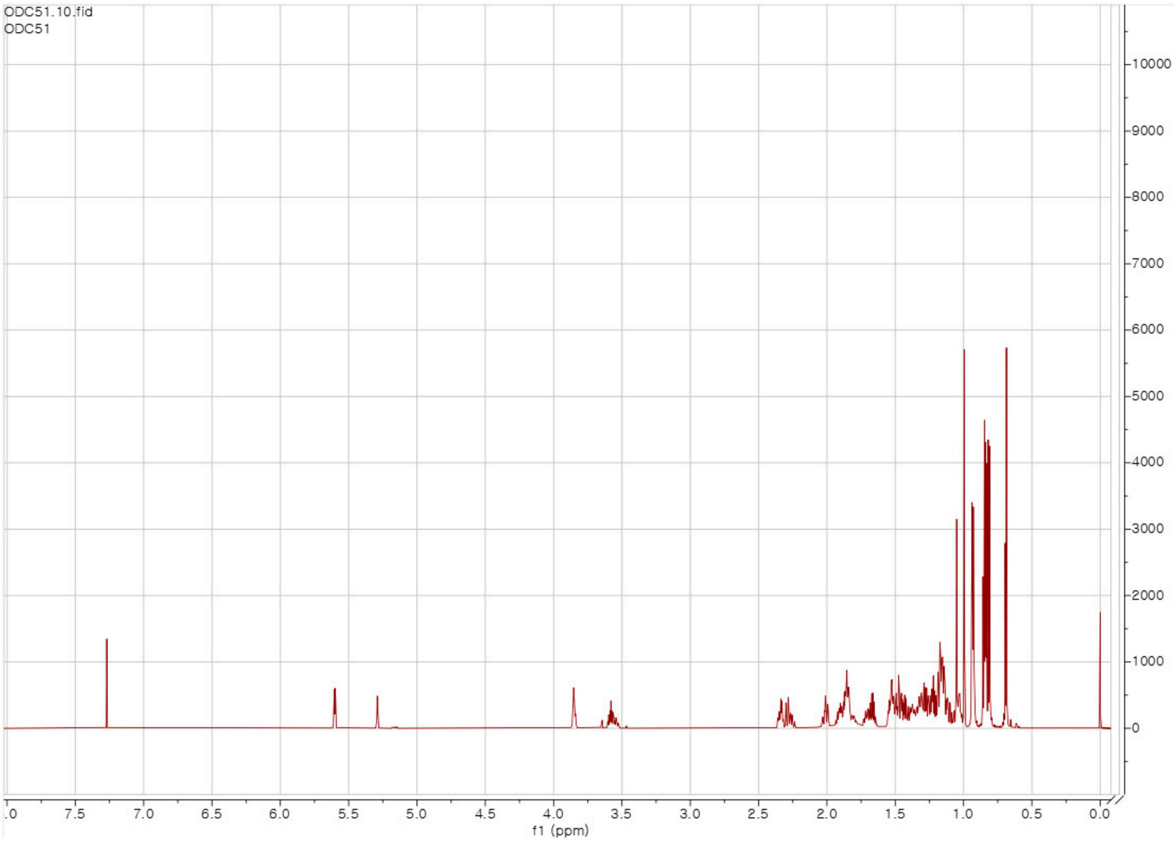

Figure S13. ^{1}H-NMR spectrum of compound **12** (in $CDCl_3$, 700 MHz)

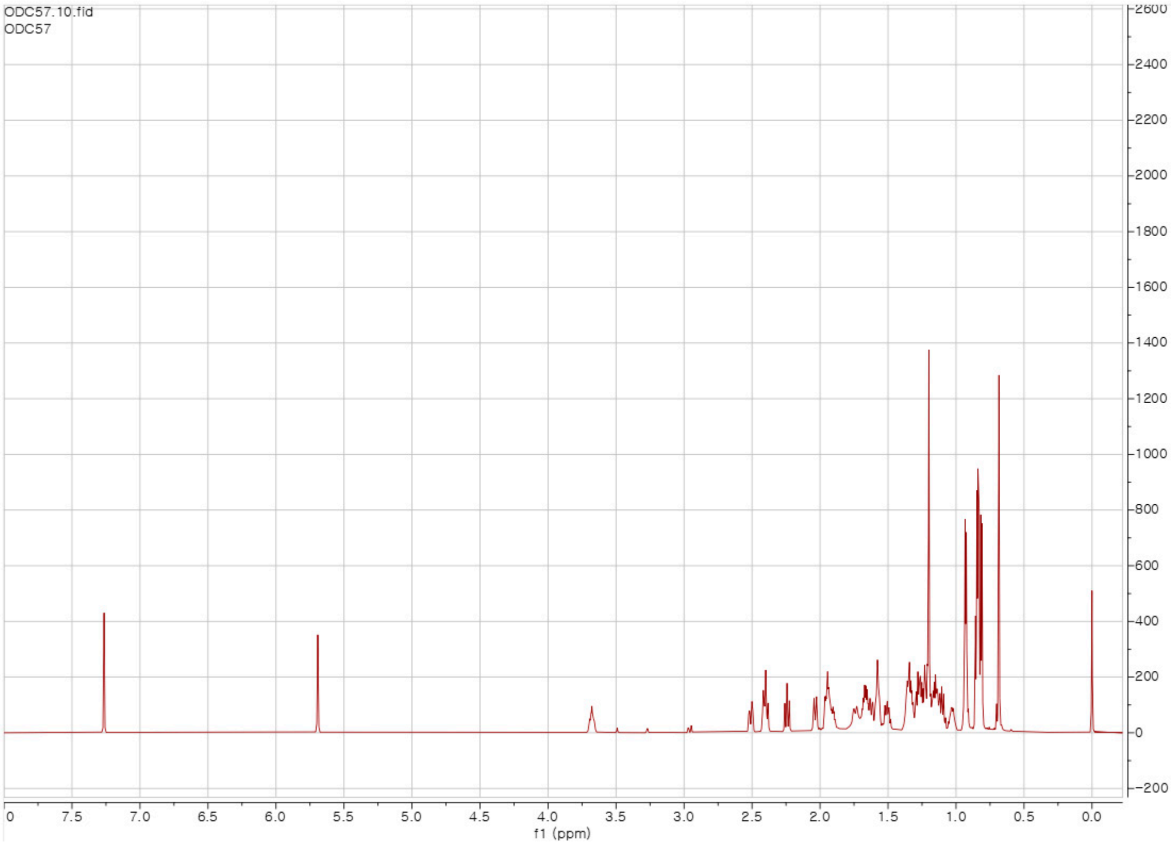

Figure S14.^{1}H-NMR spectrum of compound **13** (in $CDCl_3$, 700 MHz)

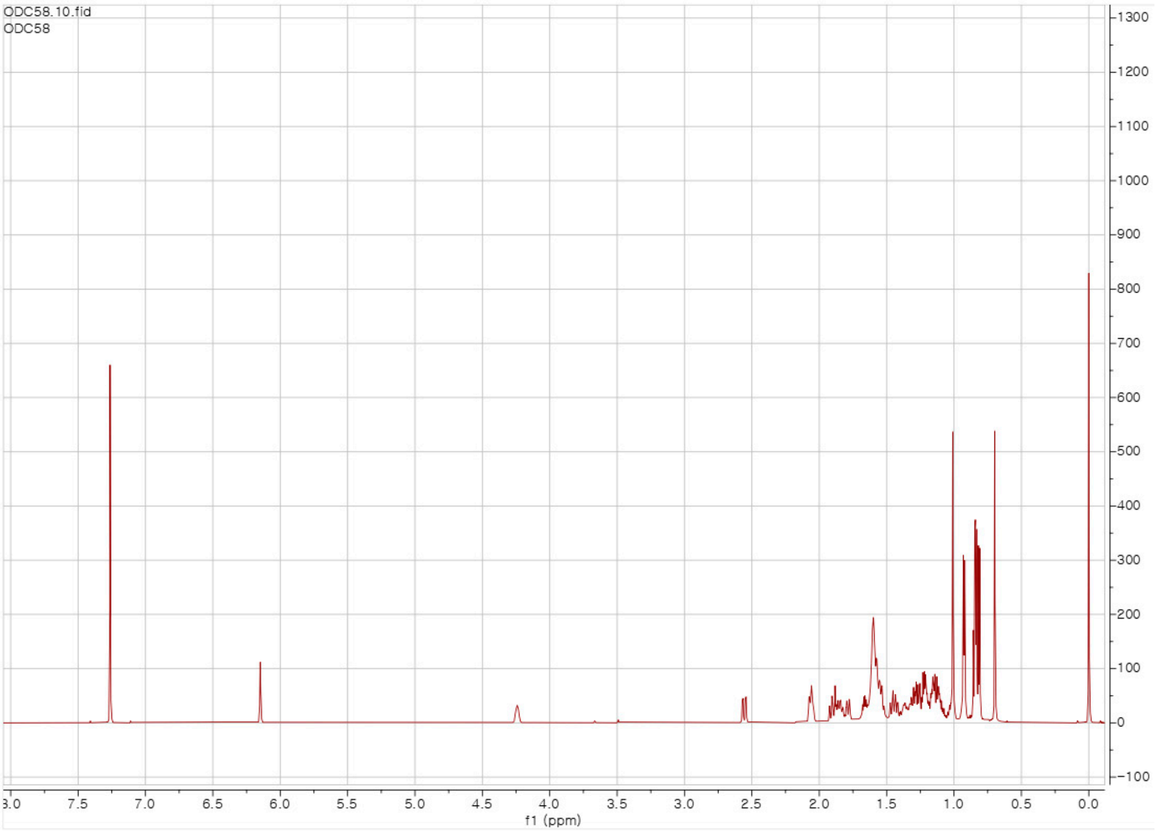

Figure S15. ^{1}H-NMR spectrum of compound **14** (in $CDCl_3$, 700 MHz)

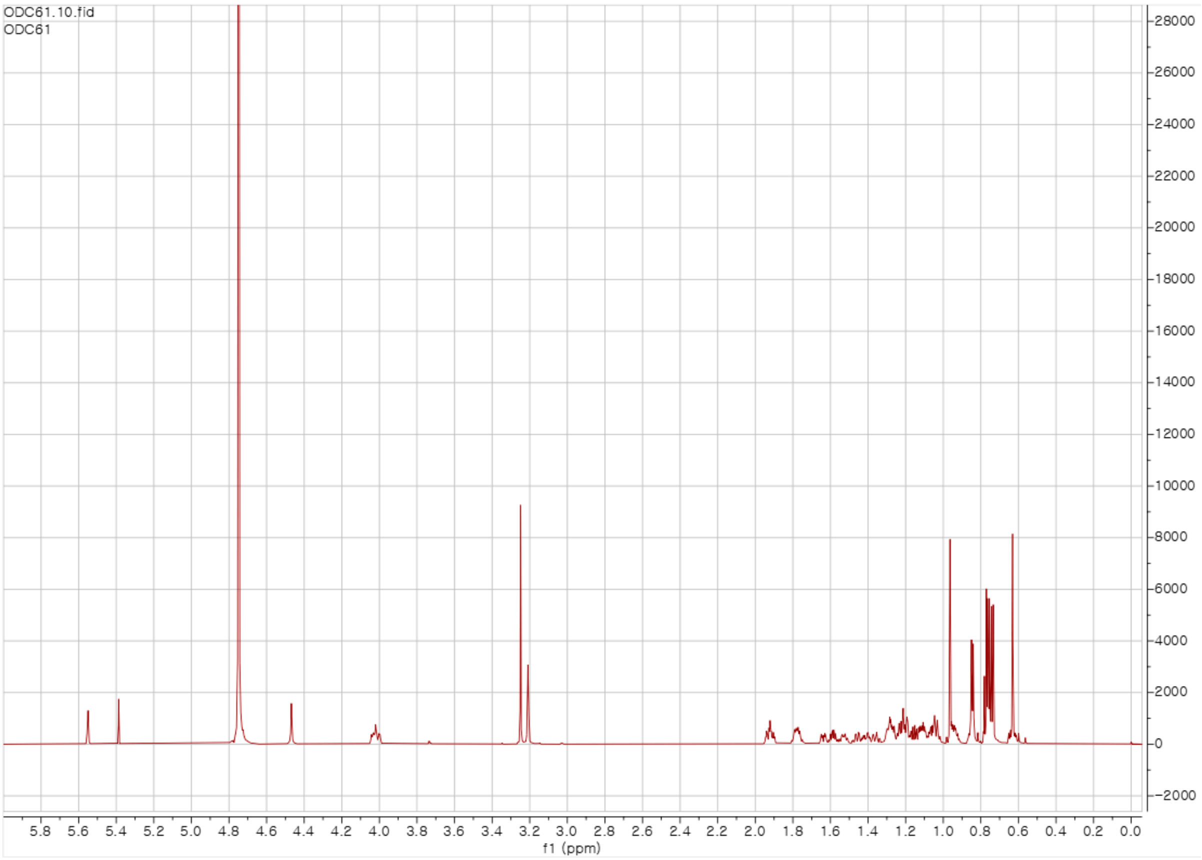

Figure S16. ^{1}H-NMR spectrum of compound **15** (in CD_3OD, 700 MHz)

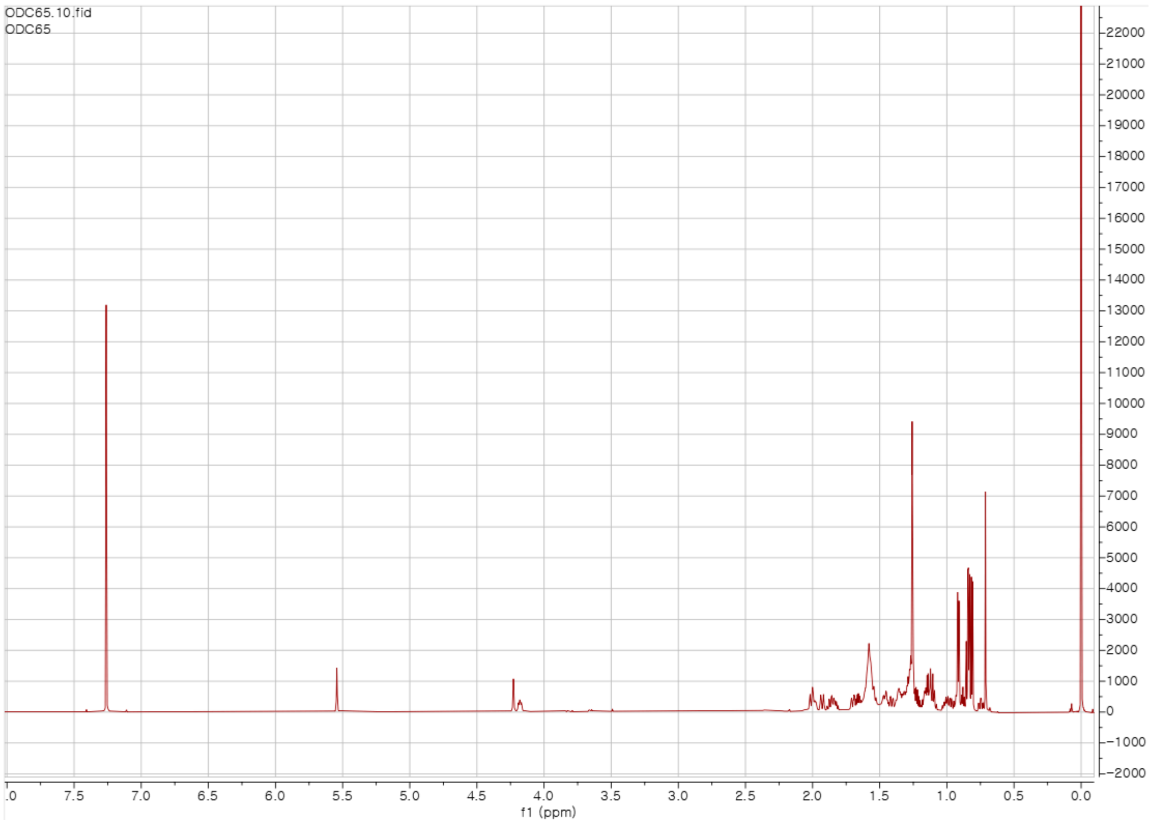

Figure S17. ^{1}H-NMR spectrum of compound **16** (in $CDCl_3$, 700 MHz)

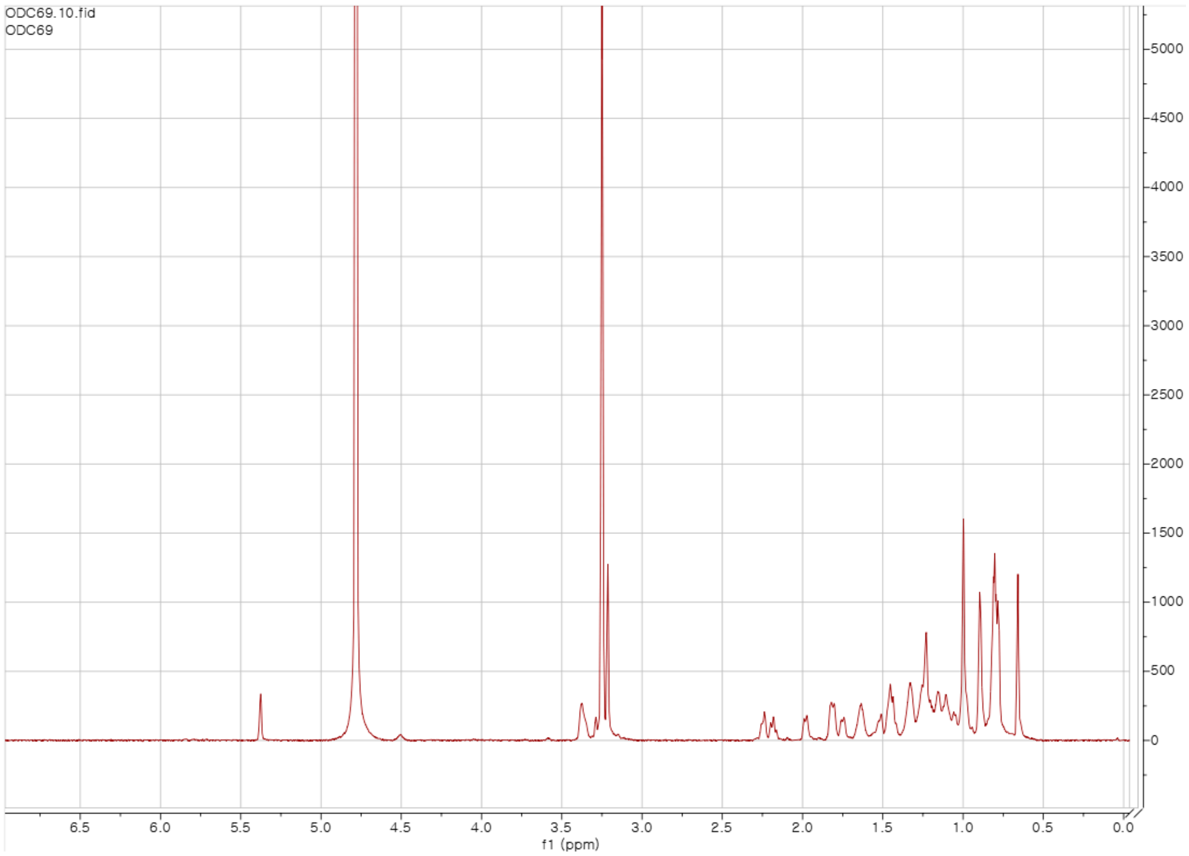

Figure S18. ^{1}H-NMR spectrum of compound **17** (in CD_3OD, 700 MHz)

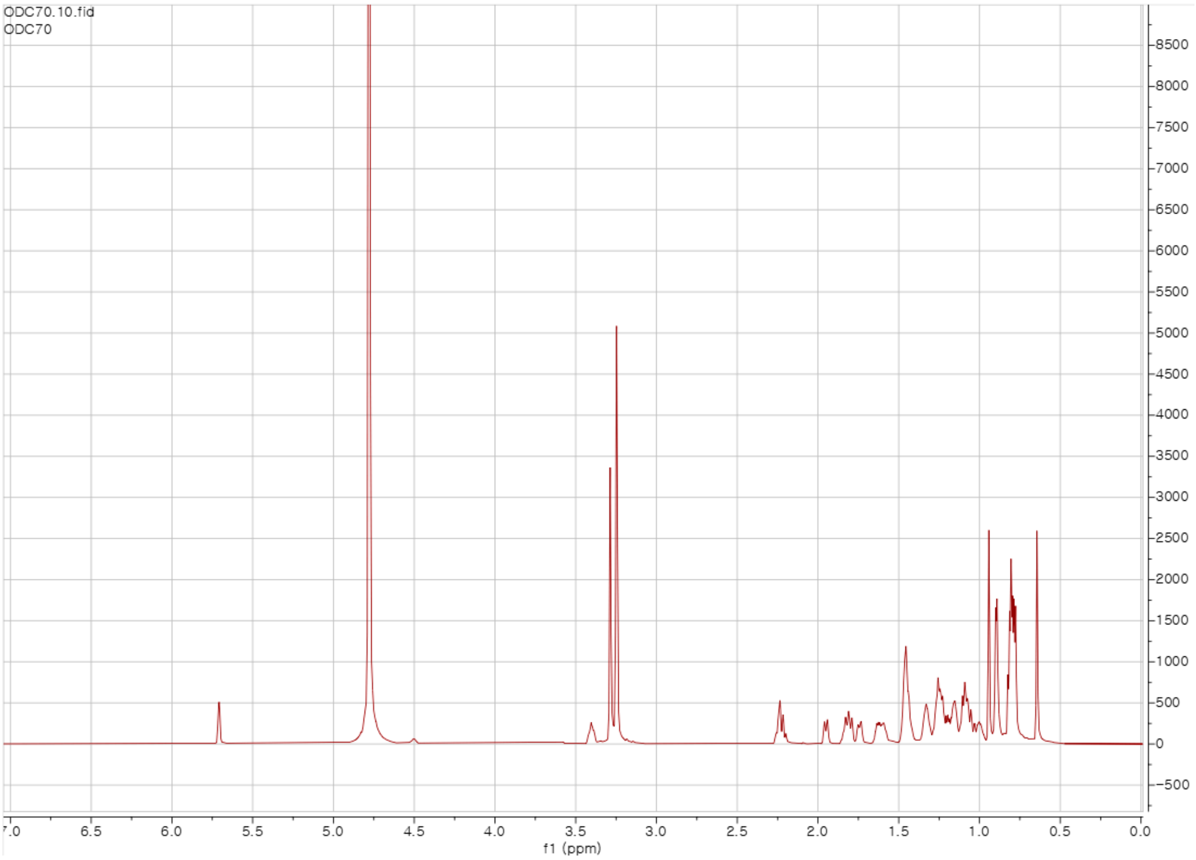

Figure S19. ^{1}H-NMR spectrum of compound **18** (in CD_3OD, 700 MHz)

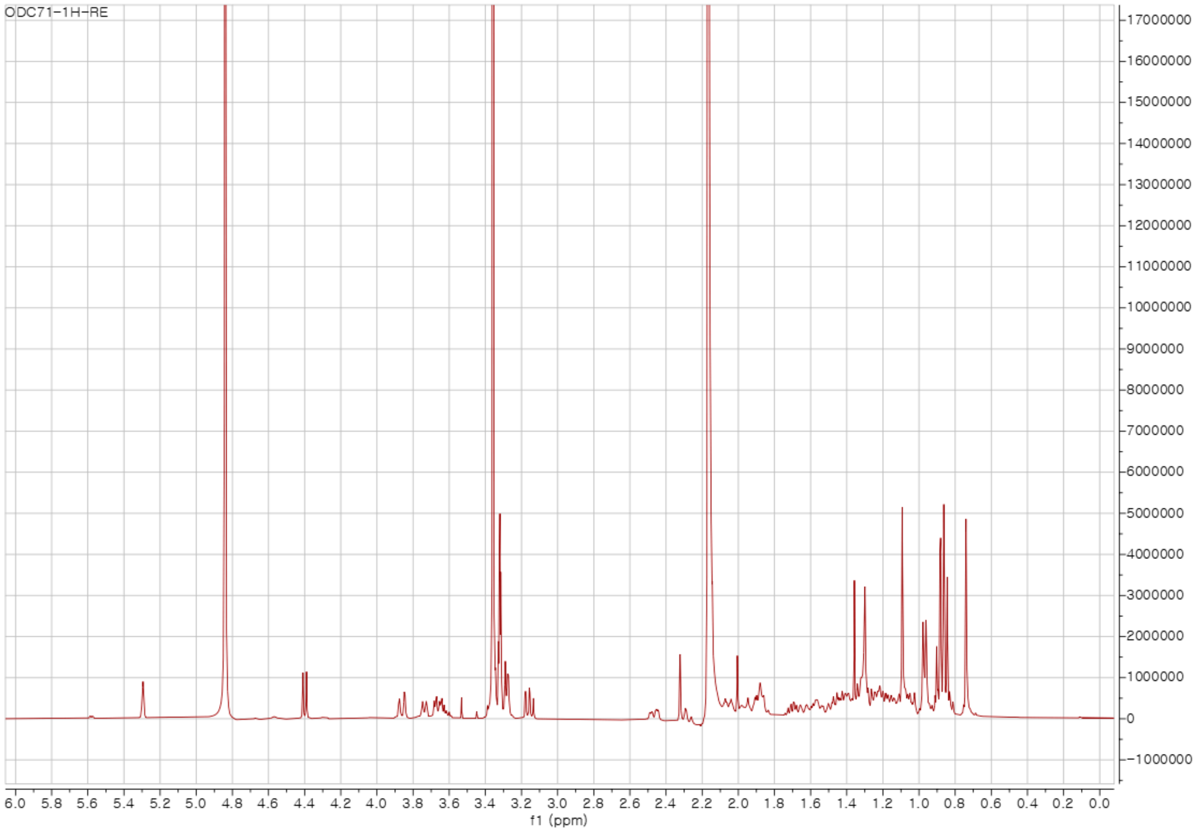

Figure S20. ^{1}H-NMR spectrum of compound **19** (in CD_3OD, 700 MHz)

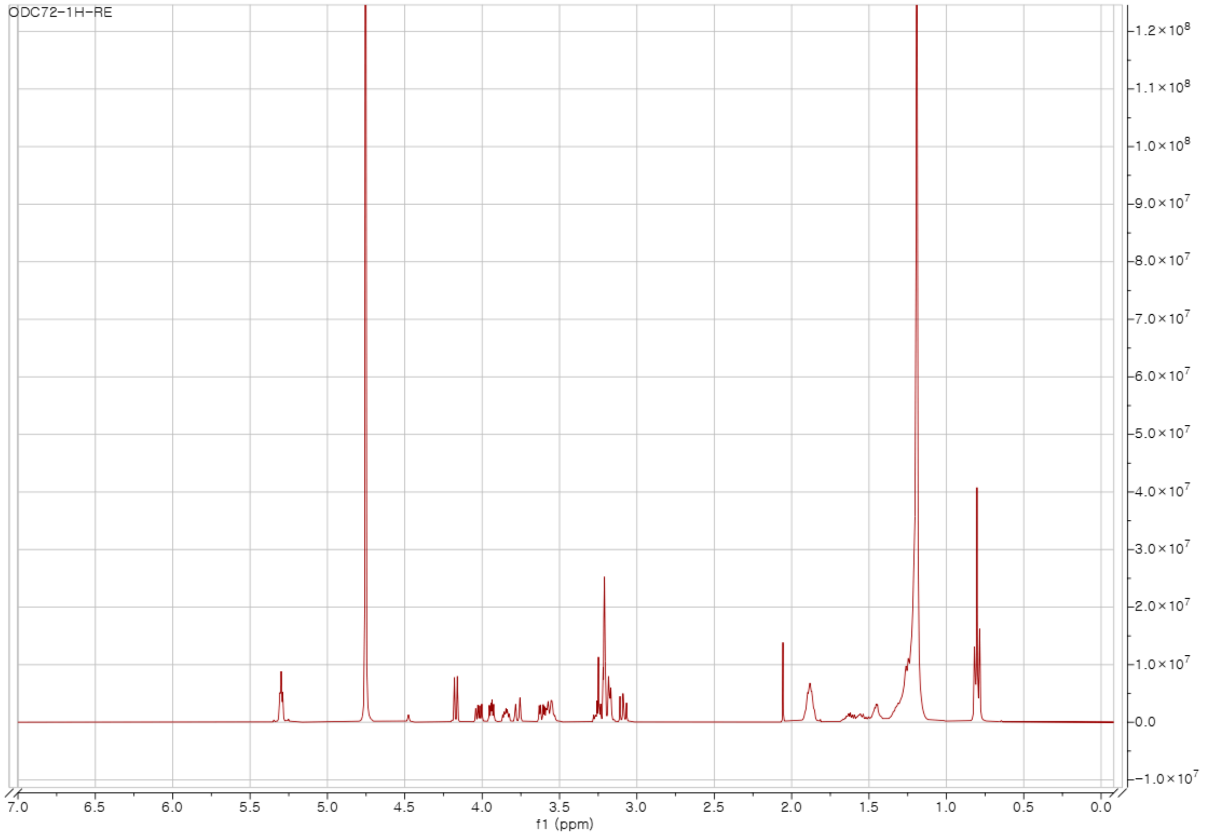

Figure S21. ^{1}H-NMR spectrum of compound **20** (in CD_3OD, 700 MHz)

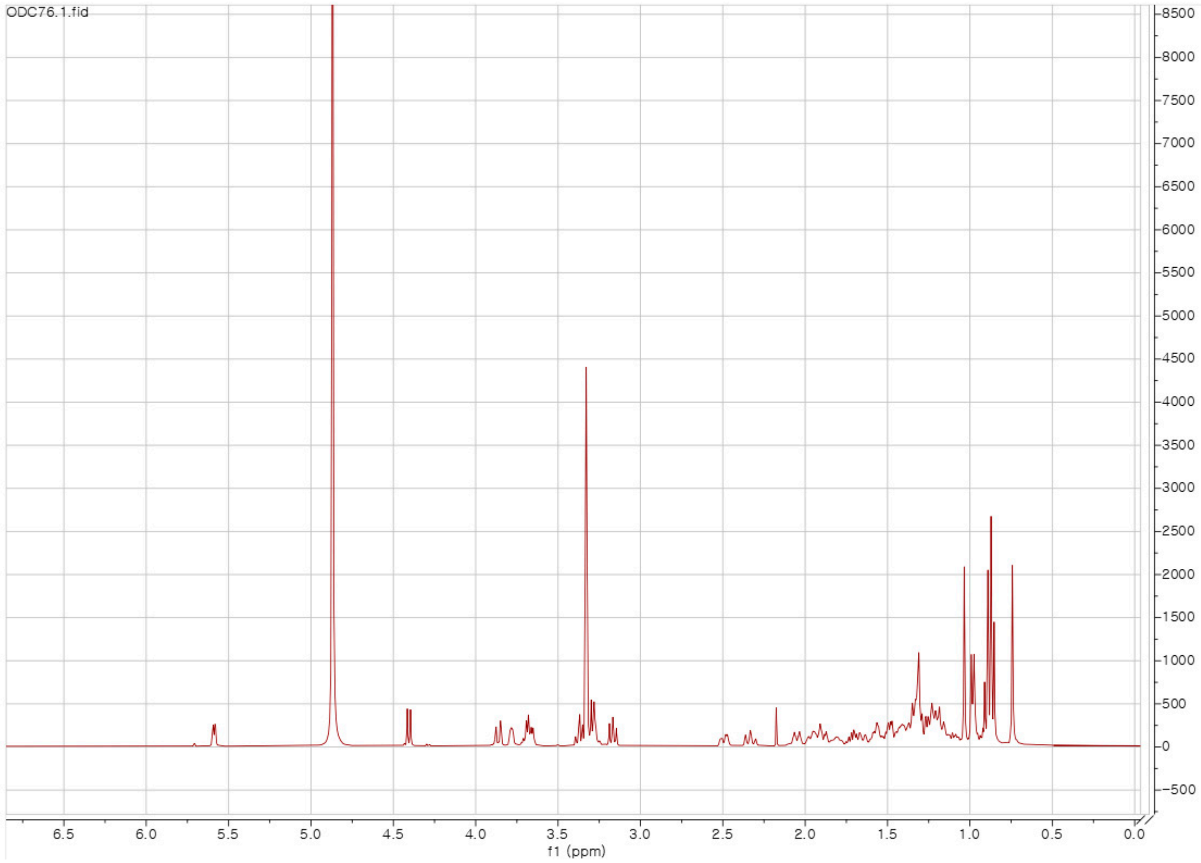

Figure S22. ^{1}H-NMR spectrum of compound **21** (in CD_3OD, 700 MHz)

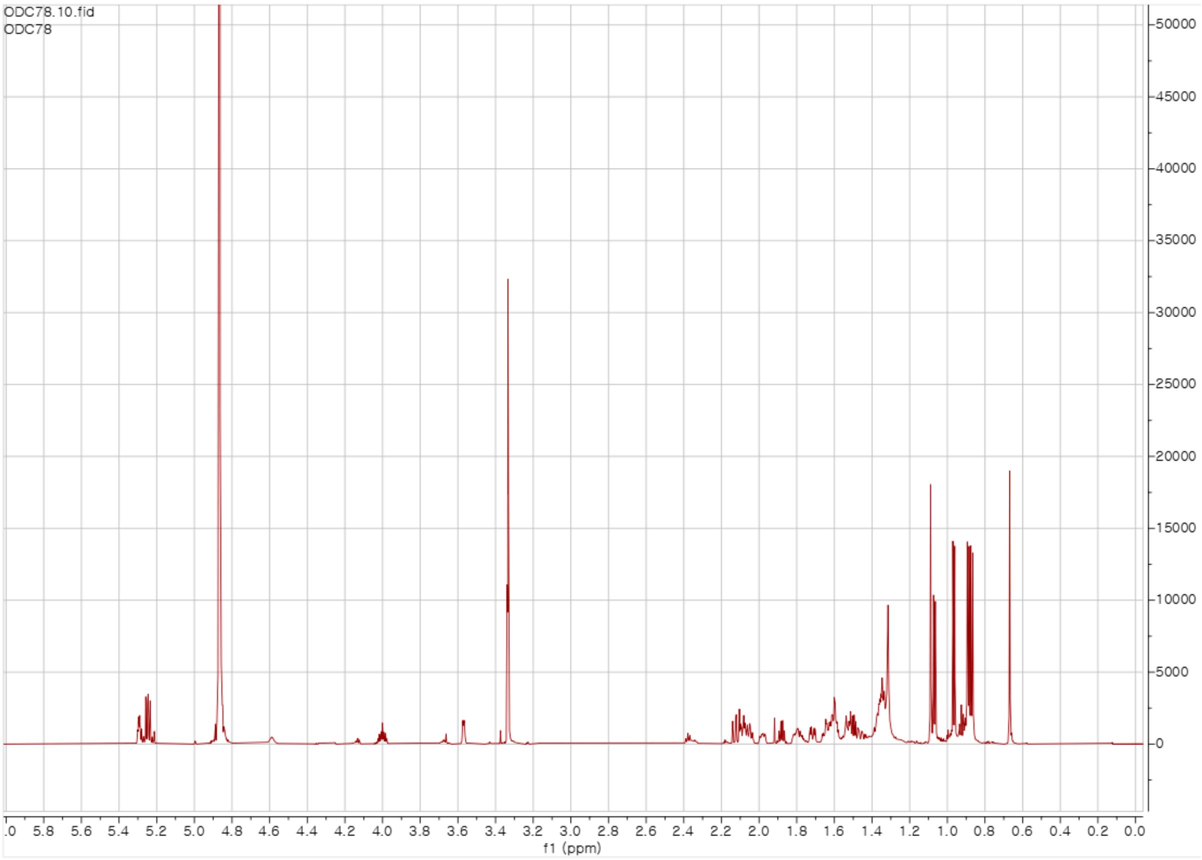

Figure S23. ^{1}H-NMR spectrum of compound **22** (in CD_3OD, 700 MHz)
